# Telomere length in relation to fecundability and use of assisted reproductive technologies: the Norwegian Mother, Father, and Child Cohort Study

**DOI:** 10.1186/s12916-024-03795-0

**Published:** 2024-12-18

**Authors:** Karoline H. Skåra, Yunsung Lee, Astanand Jugessur, Håkon K. Gjessing, Abraham Aviv, Ben Brumpton, Øyvind Næss, Álvaro Hernáez, Hans Ivar Hanevik, Per Magnus, Maria C. Magnus

**Affiliations:** 1 Centre for Fertility and Health, Norwegian Institute of Public Health, Skøyen, PO Box 222, 0213 Oslo, Norway; 2 Department of Community Medicine and Global Health, Institute of Health and Society, University of Oslo, Oslo, Norway; 3 Department of Global Public Health and Primary Care, University of Bergen, Bergen, Norway; 4 Center of Human Development and Aging, New Jersey Medical School, Rutgers University, Newark, NJ USA; 5 HUNT Center for Molecular and Clinical Epidemiology, Department of Public Health and Nursing, NTNU, Norwegian University of Science and Technology, Trondheim, Norway; 6 Department of Public Health and Nursing, HUNT Research Centre, NTNU, Norwegian University of Science and Technology, 7030 Levanger, Norway; 7 Clinic of Medicine, St. Olavs Hospital, Trondheim University, Trondheim, Norway; 8 Division of Mental and Physical Health, Norwegian Institute of Public Health, Oslo, Norway; 9 Blanquerna School of Health Sciences, Universitat Ramon Llull, 08025 Barcelona, Spain; 10 Consortium for Biomedical Research—Pathophysiology of Obesity and Nutrition (CIBEROBN), Instituto de Salud Carlos III, Monforte de Lemos 3-5, 08029 Madrid, Spain; 11 Telemark Hospital Trust, Fertilitetsavdelingen Soer, Porsgrunn, Norway

**Keywords:** Assisted reproductive technologies, Fecundability, Infertility, Mendelian randomization, MoBa, MRBN, Telomere length

## Abstract

**Background:**

Telomere length (TL) has been reported to be associated with conditions such as endometriosis and polycystic ovary syndrome, with some studies finding associations with shorter TL and others with longer TL. In men, studies mostly report associations between shorter TL and sperm quality. To our knowledge, no studies have thus far investigated associations between TL and fecundability or the use of assisted reproductive technologies (ART).

**Methods:**

This study is based on the Norwegian Mother, Father, and Child Cohort (MoBa) Study and uses data from the Medical Birth Registry of Norway (MBRN). We included women (24,645 with genotype data and 1054 with TL measurements) and men (18,339 with genotype data and 965 with TL measurements) participating between 1998 and 2008. We investigated associations between leukocyte TL (LTL) and fecundability (defined as the probability to conceive within a given menstrual cycle), infertility (defined has having spent 12 months or more trying to conceive without success), and ART use. We also repeated the analyses using instrumental variables for LTL consisting of genetic risk scores for LTL and genetically predicted LTL.

**Results:**

Approximately 11% of couples had experienced infertility and 4% had used ART. LTL was not associated with fecundability in women (fecundability ratio [FR], 0.98; 95% confidence interval [CI], 0.92–1.04) or men (FR, 0.99; CI, 0.93–1.06), nor with infertility in women (odds ratio [OR], 1.03; CI, 0.85–1.24) or men (OR, 1.05; CI, 0.87–1.28). We observed an increased likelihood of using ART with increasing LTL in men (OR, 1.22; CI, 1.03–1.46), but not in women (OR, 1.10; CI, 0.92–1.31). No significant associations were observed using the instrumental variables for LTL.

**Conclusions:**

We found no indication that LTL is a suitable biomarker for assessing fecundability, infertility, or ART use. Additional studies are required to replicate the association observed between LTL and ART use in men.

**Supplementary Information:**

The online version contains supplementary material available at 10.1186/s12916-024-03795-0.

## Background

Ageing entails an increased prevalence of several diseases arising from an inevitable and irreversible decline in physiological function across multiple organ systems [1]. It involves a gradual accumulation of molecular and cellular damage, including DNA mutations, oxidative stress, and telomere shortening [2]. Telomeres are DNA protein structures located at the ends of each chromosome. They consist of 5’-TTAGGG-3’ tandem repeats that serve as protective caps to prevent chromosomal degradation during DNA replication, thus maintaining genomic stability and preserving genetic information across cell divisions [3, 4]. Telomere length (TL) is commonly measured in human blood leukocytes [5], highly heritable across generations, and gradually shortens with age, triggering cellular senescence or apoptosis upon reaching a critical threshold [4, 6, 7].

Fecundability, defined as the probability of conceiving within a single menstrual cycle, declines with age [8–10]. This decline in women is largely attributable to changes such as a decrease in the number and quality of oocytes as well as altered hormonal levels [8]. Accordingly, the risk of infertility, defined as being unable to establish a clinical pregnancy after 12 months of regular, unprotected intercourse, increases with age [9]. While the impact of age on male infertility is less pronounced than it is for female infertility, there is evidence of a gradual decline in semen quality with age [11]. However, these factors only partly explain the decrease in fecundability and increase in risk of infertility with age in both sexes [12]. It has been proposed that variations in TL could explain this unexplained variation and potentially act as a biomarker for low fecundability and high risk of infertility [13, 14].

Current evidence supports an association between TL and proxies of low fecundability or infertility, such as premature ovarian failure, oocyte maturation, polycystic ovary syndrome (PCOS), and endometriosis [15, 16]. However, studies investigating these associations have produced conflicting results [15, 16]. For endometriosis, for instance, some studies report an association with longer TL [17, 18], but others with shorter TL [19, 20]. Most studies on the relationship between TL and sperm quality in men suggest that shorter TL is associated with infertility-related sperm characteristics, although some studies also report associations with longer TL [15, 21]. Generally, studies investigating the association between TL and reproductive potential involve modest sample sizes, ranging from 30 to 1200 participants [15]. Crucially, none of the above studies have specifically addressed fecundability or the use of assisted reproductive technologies (ART). Previous studies have also not incorporated the use of genetic risk scores (GRS) for TL, which could provide additional insights into the unconfounded relationship between TL and reproductive potential [22, 23]. The relationship between TL and fecundability in both women and men therefore remains unclear.

Given these important knowledge gaps, we aimed to investigate whether leukocyte TL (LTL) was associated with fecundability, infertility, or use of ART in women and men participating in the Norwegian Mother, Father, and Child Cohort Study (MoBa). Given that telomere shortening can potentially impair cellular function, affecting key reproductive processes such as egg and sperm quality, ovarian reserve and overall infertility, we hypothesized that there would be an association between LTL and fecundability, infertility, and ART use in both women and men.

## Methods

### Study population

We studied participants in MoBa, a population-based pregnancy cohort in which pregnant women and their partners were recruited around the 17th week of gestation between 1998 and 2008 [24, 25]. Blood samples were collected at recruitment, and the majority of the participants have been genotyped [26, 27].

**Fig. 1:**
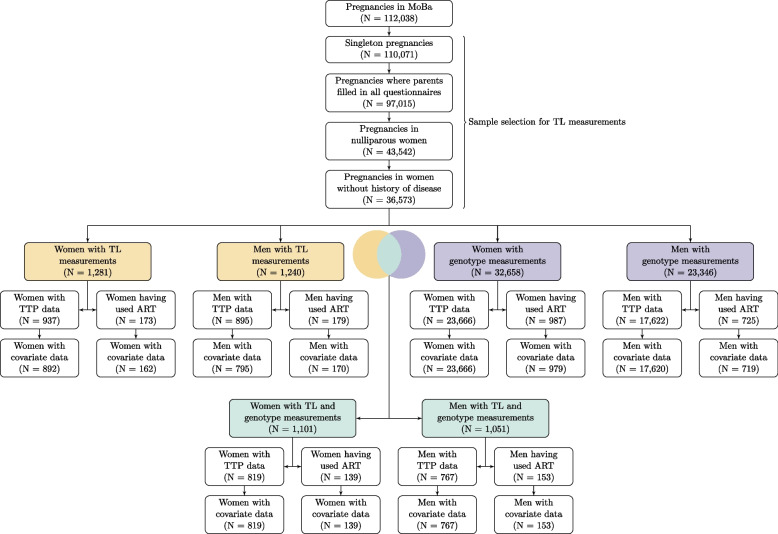
Flowchart of the study population. Shown here are the participants for whom we had (i) only leukocyte telomere length (LTL) measurements (in yellow), (ii) only genotypes (in purple), and (iii) both LTL measurements and genotypes (in green)

Measurements of LTL were conducted on a subset of nulliparous women and their partners who had term singleton live births and responded to questionnaires administered at gestational weeks 17 and 30. Couples in which the women had a history of chronic hypertension or diabetes, as well as those registered with preeclampsia during pregnancy or with a baby diagnosed with congenital anomalies, were excluded from the LTL measurements (see Fig. 1). For the present study, we only included couples who reported their time to pregnancy (TTP) and those who reported having conceived using ART.

The Regional Committee for Medical and Health Research Ethics of South-East Norway (REK 2017/1362) approved this study. A written informed consent was obtained from all participants. To facilitate comparisons between studies, our work adheres with the “Strengthening the Reporting of Observational Studies in Epidemiology (STROBE)” guidelines for reporting Mendelian randomization and cohort studies.

### Telomere measurements

Average LTL was measured in 1597 women and 1582 men using the Southern blotting method, which is based on measuring terminal restriction fragments (TRFs) twice and taking the mean of the two measurements as previously described [28]. After applying the selection criteria mentioned above, pregnancies were randomly selected for the study and classified according to ART use. For studying ART, LTL was only measured in women aged 30 years or older, as women who use ART tend to be older and above this age cutoff. In contrast, in women conceiving through sexual intercourse, LTL was measured in women aged 18 years or above, with a targeted oversampling of women at about 32 years of age (Additional file 1: Fig. S1). Given the strong correlation between TL and age due to the gradual attrition of TL with each cell division, we calculated residual LTL by regressing LTL against the age at which LTL was measured to obtain age-adjusted LTL for all analyses (hereafter referred to simply as LTL; see Additional file 1: Fig, S2).

### Genetics of telomeres

Genotyping of MoBa samples was carried out in 26 separate batches, each with varying selection criteria, genotyping arrays, genotyping core facilities, and specific quality control (QC) criteria used by the “MoBaPsychGen” pipeline as outlined in Corfield et al. [29]. For variant calling, single-nucleotide polymorphisms (SNPs) with a minor allele frequency (MAF) of < 0.5%, call rate < 98%, Hardy–Weinberg equilibrium *p*-value < 1 × 10^–6^, or heterozygosity within ± 3 standard deviations from the mean were excluded. Samples were also excluded for erroneous sex assignment (i.e., genetically inferred sex contradicting reported sex). The European Genome-Phenome Archive Haplotype Reference Consortium (HRC) release 1.1 was used as the reference panel for both pre-phasing and imputation. Post-imputation QC was conducted on the merged imputation batches, retaining only SNPs that passed QC in all batches (see Corfield et al. [29] for further details).

In order to minimize the impact of unmeasured confounding, which often bias observational studies, and enhance statistical power in our study, we calculated GRS as instrumental variables for LTL based on the framework for one-sample Mendelian randomization analyses [30]. We identified independent SNPs significantly associated with LTL (*P* < 5 × 10^–8^) from the most recent genome-wide association study (GWAS) of LTL by Codd et al. [31]. Of the 197 SNPs identified by Codd et al. [31], only 120 were present in our MoBa genotype dataset. To handle the missing SNPs, we searched for substitute SNPs in strong linkage disequilibrium with the missing SNPs (*R*^2^ > 0.9) within a 1-Mb window of the missing SNPs, allowing us to include 144 SNPs for computing GRS.

The GRS for LTL were calculated by summing up the weighted risk alleles using effect sizes from Codd et al. [31] and applying the formula: $\text{GRS}= \sum_{i=1}^{m}{\beta }_{i}{\text{SNP}}_{i}$, where ${\beta }_{i}$ represents the effect of the $i$ th SNP, *m* the number of SNPs showing associations as risk predictors, and ${SNP}_{i}$ the number of effect alleles for the $i$ th SNP. We also used the GRS to estimate genetically predicted age-adjusted LTL through two-stage least square (2SLS) regression, adjusting for the first ten ancestry-informative genetic principal components, as outlined by Burgess et al. [32].

### Fecundability, infertility, and use of assisted reproductive technologies

We used self-reported TTP as a measure of fecundability, defined as the probability of conceiving during a given menstrual cycle. Women indicated whether their pregnancy was planned and, if so, the time spent trying to conceive. For this variable, the options were “ < 1 month,” “1–2 months,” or “3 or more months”. We assigned a TTP value of 1 and 2 months to the first two categories, respectively. For those reporting “3 or more months,” the exact number of months spent trying to conceive was used as their TTP. If the exact number of months was not provided, a TTP value of 3 months was assigned. For women who reported their cycle length, TTP was adjusted to reflect the number of cycles rather than the number of months. To investigate infertility specifically, we classified couples as experiencing infertility if they had tried to conceive for at least 12 months before succeeding, based on the women’s self-reports [9]. Couples who did not have a planned pregnancy, used contraceptives at conception, or conceived through ART were excluded from this analysis.

Information on the use of ART, the main and contributing reasons for using ART, and ART treatment modality was obtained through linkage with the Medical Birth Registry of Norway. As a sub-analysis of the main reason for ART use, women registered for having endometriosis, ovulatory disorders, or tubal factor infertility as the main reason for ART use were classified as “main female-factor infertility”. Men registered for having sperm factor infertility as the main reason for ART use were classified as “main male-factor infertility”. Similarly, as a sub-analysis of contributing reasons to ART use, women registered for having endometriosis, ovulatory disorders, tubal factor infertility, or uterus anomalies as contributing reasons for ART use were classified as “contributing female-factor infertility”. Similarly, men registered for having sperm factor infertility as a contributing reason for ART use were classified as “contributing male-factor infertility”.

### Statistical analyses

To examine the effect of LTL on fecundability, infertility, and the use of ART, we examined the three measures of LTL separately, i.e., (i) LTL measured by Southern Blot (hereafter referred to as TRF-determined LTL), (ii) GRS for TL, and (iii) genetically predicted TL. For the analysis of fecundability, we further used proportional probability regression and a discrete survival approach to estimate fecundability ratios (FRs), using the various LTL measures as exposures and menstrual cycles as the unit of time in each analysis. This allowed us to estimate the relative difference in the probability of conceiving within a given menstrual cycle, according to increasing levels of the three LTL measures. This approach assumes that there is no disproportionate effect of any of the variables on the probability to conceive within a specific cycle. We censored the TTP at twelve cycles as this is when couples are more prone to seek infertility treatment. We also investigated the possibility of non-linear associations between the various LTL measures and fecundability using generalized additive models (GAMs) with restricted cubic splines using the *mgcv* R package [33]. We assessed model fit based on the effective degrees of freedom (EDF) and the Akaike information criterion (AIC) [33]. For the analyses of differences in propensity to infertility and ART use according to increasing levels of the three LTL measures, we used logistic regression to estimate the odds ratio (OR) for each of these outcomes.

The analyses of TRF-determined LTL were further adjusted for age (continuous), pre-pregnancy body mass index (BMI; continuous; kg/m^2^), highest completed or ongoing education level (categorical; university and high school or below), and smoking status (categorical; non-smoker, former smoker, and smoker during the last 3 months before pregnancy). In the analyses using GRS for LTL and genetically predicted LTL as exposures, we adjusted for age (continuous) and the first five genomic principal components (continuous). When analysing GRS for LTL, we used age at birth for the MoBa index pregnancy as an alternative to the age when the TRF-determined LTL was measured. To ensure that these measures are comparable, we investigated the associations with fecundability, infertility, and ART use per standard deviation (SD) increase in the LTL measures.

### Sensitivity analyses

Since LTL was only measured in women aged 30 years or older and their partners in the ART group, we conducted sensitivity analyses excluding women and men under 30 from the non-ART comparison (the reference group) in the analyses of ART use, as well as from all analyses of fecundability and infertility. Given that a couple’s LTL can be correlated due to assortative mating, we carried out another sensitivity analysis where we mutually adjusted for the LTL measures of partners.

### Software

Analyses were performed in R software version 4.2.3 [34, 35].

## Results

Our study population included 1054 women and 965 men in the analysis of TRF-determined LTL. Overall, 24,645 women and 18,339 men were included in the analysis of GRS for LTL and 958 women and 920 men in the analysis of genetically predicted LTL (Fig. 1). Among those assessed for TRF-determined LTL and genetically predicted TL, 15% of couples had spent 12 months or more trying to conceive, whereas approximately 20% of couples had used ART to conceive. The mean age within this subsample was 32 years (SD = 4 years) for women and 34 years (SD = 5 years) for men. Within the subsample of the study population with data on GRS for LTL, 11% of couples had spent 12 months or more trying to conceive, whereas 4% of couples had used ART to conceive. The mean age among these individuals was 29 years (SD = 4 years) for women and 32 (SD = 5 years) for men.

Across all groups, those with infertility who conceived through sexual intercourse or ART typically had a slightly higher BMI than those who spent less than 12 months to conceive but were otherwise similar with respect to educational level and smoking behaviour (Table 1, Additional file 1: Table S1). The Pearson correlation coefficient for TRF-determined LTL between partners was 0.31 (Additional file 1: Fig. S3). The GRS for LTL and genetically predicted LTL were associated with longer TRF-determined LTL in both sexes, explaining 6% of TRF-determined LTL variation (Pearson correlation coefficient = 0.25, Additional file 1: Fig. S5 and S6). Using the first-stage F-statistic to test the strength of the association between GRS and TRF-determined LTL, we found no signs of weak instrument bias (F-statistic > 10, Additional file 1: Fig. S5 and Table S2) [36].

**Table 1:** Characteristics of the study population

	**Women**			**Men**		
	**Fertile**	**Infertile**	**ART use**	**Fertile**	**Infertile**	**ART use**
N (%)	784	153	173	753	142	179
Age, median (IQR)	32.1 (28.6, 33.8)	33.3 (31.7, 34.8)	34.1 (32.4, 35.9)	32.7 (29.9, 35.8)	34.0 (31.1, 37.8)	35.8 (33.6, 38.8)
TL, median (IQR)	7.8 (7.3, 8.3)	7.7 (7.3, 8.1)	7.9 (7.3, 8.3)	7.7 (7.2, 8.1)	7.7 (7.3, 8.1)	7.8 (7.3, 8.3)
TTP, median (IQR)	2 (2, 5)	17 (12, 26)		3 (2, 5)	17 (12, 28)	
BMI, median (IQR)	22.8 (21.1, 25.3)	23.7 (21.3, 27.5)	22.9 (21.4, 25.1)	25.4 (23.5, 27.7)	25.7 (24.0, 27.6)	25.6 (24.3, 27.8)
BMI, N (%)	770 (98.2)	153 (100.0)	171 (98.8)	740 (98.3)	139 (97.9)	177 (98.9)
Missing, N (%)	14 (1.8)	0 (0.0)	2 (1.2)	13 (1.7)	3 (2.1)	2 (1.1)
Higher education, N (%)	579 (73.9)	110 (71.9)	148 (85.6)	455 (60.4)	85 (59.6)	120 (67.0)
Lower education, N (%)	187 (23.9)	39 (25.5)	17 (9.8)	282 (37.9)	57 (40.1)	57 (31.9)
Missing, N (%)	18 (2.3)	4 (2.6)	8 (4.6)	13 (1.7)	0 (0.0)	2 (1.1)
Non-smoker, N (%)	406 (51.8)	78 (51.0)	90 (52.0)	297 (39.4)	55 (38.7)	66 (36.9)
Smoker > 3 mo. ago, N (%)	158 (20.2)	29 (19.0)	45 (26.0)	192 (25.5)	36 (25.4)	59 (33.0)
Smoker last 3 mo., N (%)	212 (27.0)	45 (29.4)	36 (20.8)	192 (25.5)	45 (31.7)	48 (26.8)
Missing, N (%)	8 (1.0)	1 (0.6)	2 (1.2)	72 (9.6)	6 (4.2)	6 (3.4)

Characteristics of the participants in the study population with measurements of telomere length

### Fecundability and infertility

**Fig. 2:**
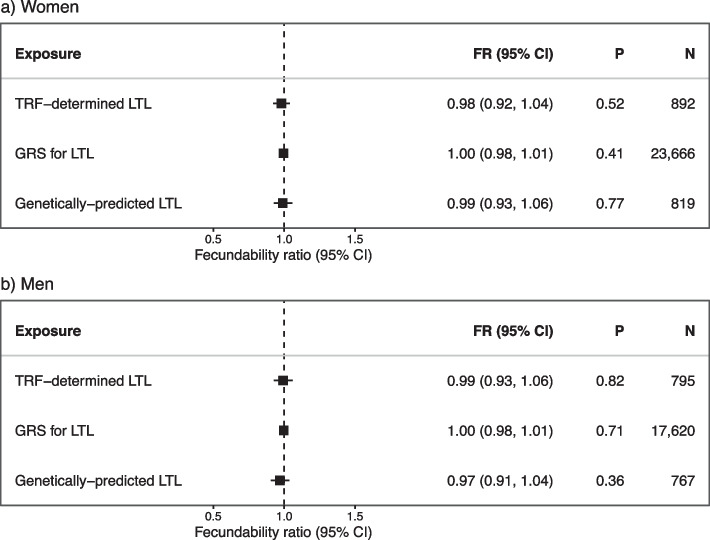
The associations between telomere length and fecundability. The associations between one standard deviation (SD) increase in TRF-determined leukocyte telomere length (LTL) measures and fecundability in **a** women and **b** men

**Fig. 3:**
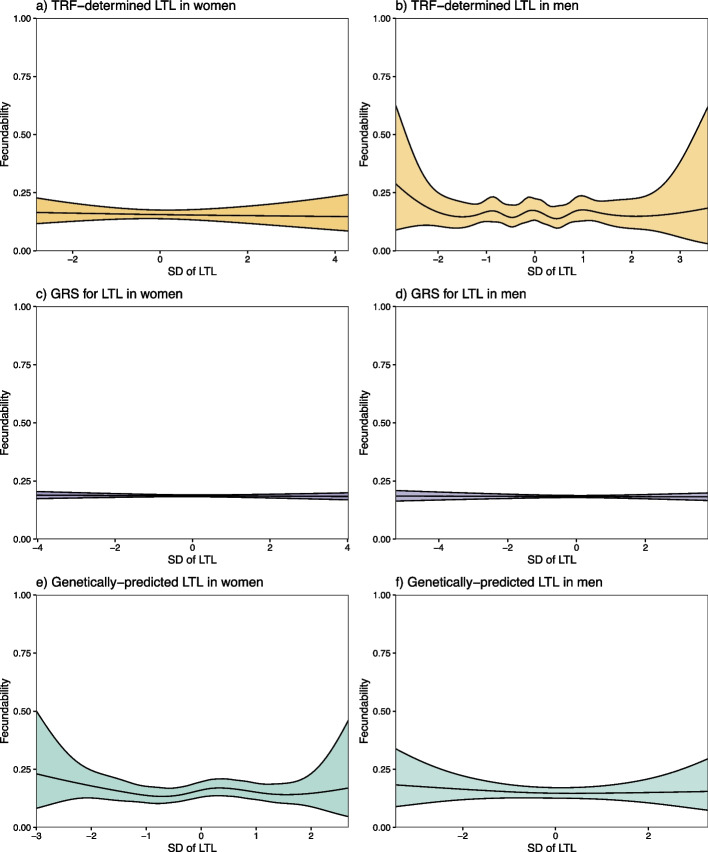
Non-linear associations between telomere length and fecundability. The non-linear associations between one standard deviation (SD) in TRF-determined leukocyte telomere length (LTL) for **a** women and **b** men, genetic risk scores (GRS) for LTL for **c** women and **d** men, and genetically predicted LTL for **e** women and **f** men, in relation to fecundability. The colour scheme is the same as in the flowchart in Fig. 1

**Fig. 4:**
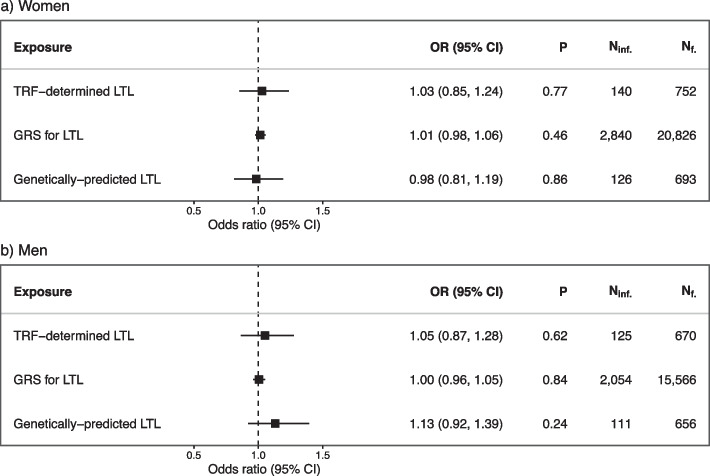
The associations between telomere length and infertility. The associations between one standard deviation (SD) increase in TRF-determined leukocyte telomere length (LTL) measures and infertility for **a** women and **b** men

We found no significant associations between any of the LTL measures and fecundability in both women and men (Fig. 2). The effects observed were mostly proportional to each other across menstrual cycles (Additional file 1: Fig. S7 and S8). Furthermore, there was no strong evidence of any non-linear relationships between LTL and fecundability across the different LTL measures (Fig. 3, Additional file 1: Table S3). Similarly, we found no significant associations between any of the LTL measures and infertility in either sex (Fig. 4).

### Assisted reproductive technologies

**Fig. 5:**
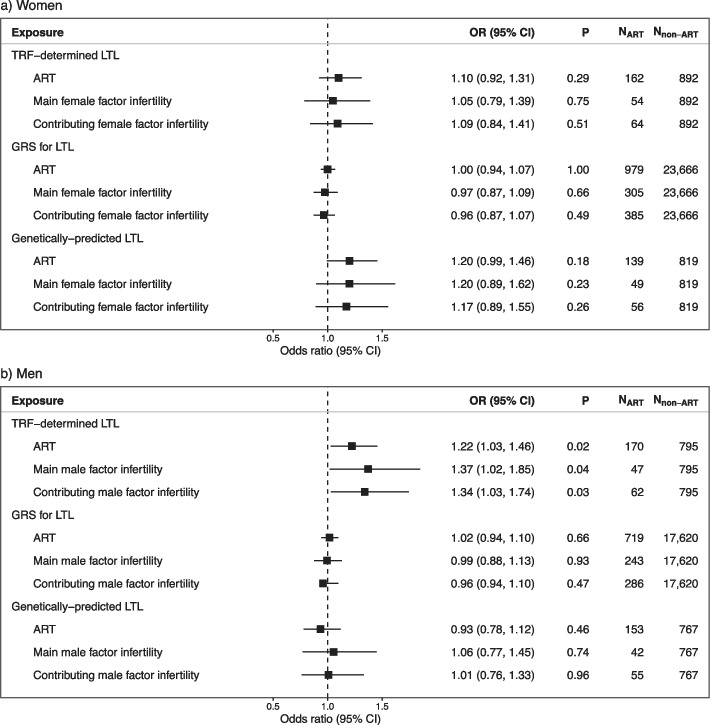
The associations between telomere length and use of assisted reproductive technologies. The association between a standard deviation (SD) increase in TRF-determined leukocyte telomere length (LTL) measures and having conceived through assisted reproductive technologies (ART) in **a** women and **b** men

We found no significant associations between any of the LTL measures and ART use in women (Fig. 5). In men, however, we found an association between longer TRF-determined LTL and increased risk of ART use (OR, 1.22; 95% confidence interval [CI], 1.03–1.1.46). The latter result was consistent when investigating both male factor infertility as the main reason for using ART (OR, 1.37; CI, 1.02–1.85) and as any reason for using ART (OR, 1,34; CI, 1.03–1.74). However, the increased likelihood of ART use with longer LTL in men was not observed when investigating GRS for LTL and genetically predicted LTL.

### Sensitivity analyses

The results remained consistent when we restricted the study population to women and men aged 30 years or older in the analyses of fecundability, infertility, and ART use (Additional file 1: Fig. S9, S10 and S11) and when adjusting for partners’ LTL measures in all analyses (Additional file 1: Fig. S12, S13 and S14).

## Discussion

To our knowledge, this is the first study to investigate the relationship between LTL, fecundability, and ART use. In this large population-based study of healthy women and men who were able to conceive, we found no significant associations between LTL measures and fecundability or infertility. However, we found a higher likelihood of ART use in men with longer TRF-determined LTL, a pattern not found in women. This observed association in men using ART persisted, regardless of whether male factor infertility was considered as a main reason, when it was considered a contributing reason for ART use, or when mutually adjusting for their partners’ TRF-determined LTL. Curiously, this increased likelihood of ART use in men with longer LTL was not replicated when we used GRS for LTL or genetically-predicted LTL.

Our null findings contrast with those of earlier studies reporting associations between TL and infertility-related phenotypes in women, such as PCOS and endometriosis, as well as an association between TL and sperm quality in men [15, 16, 21]. However, as studies have identified associations with both shorter and longer TL compared to those without infertility-related phenotypes, these mixed results highlight the ambiguity in interpreting any potential relationship between TL and reproductive potential. For example, our findings may have been influenced by the study being restricted to couples who eventually conceived, carried a pregnancy to term, and had a live birth. This could have affected our analyses. Additional studies of TL that focus on couples who remain infertile would be valuable. Moreover, as TRF-determined LTL was measured during pregnancy in our study, it is possible that pregnancy itself might have influenced LTL in women. For instance, endogenous oestrogens might be associated with longer TL [37], and pregnancy could potentially alter TL in women. However, a recent review showed that TL did not change markedly during early pregnancy [38]. Whether pregnancy affects TL directly remains unclear. Furthermore, hormone treatments administered to women during ART procedures to stimulate ovulation may have impacted the LTL measures. However, any association detected between TRF-determined LTL and ART use in men cannot be attributed to hormone treatments, as men are not subjected to such treatment.

The significant association between longer TRF-determined LTL and ART use in men in our study contradicts previously reported associations between shorter TL and sperm-related factors. However, several studies have identified a positive correlation between offspring’s LTL and paternal age at conception (PAC), even after adjusting for offspring’s age [39–42]. This ‘PAC effect’ could potentially explain the observed association between TRF-determined LTL and ART use in men. It has been proposed that TL in sperm cells increases with age and that a more robust telomerase activity in these cells could be a mechanism for the observed PAC effect [42]. Moreover, since older men have lower sperm counts, the amount of telomerase available per sperm cells is also greater in older men [43]. Consequently, the offspring of older fathers tend to have longer LTL. To examine this further, we calculated paternal residual LTL by regressing the TRF-determined LTL on the fathers’ age when they themselves were born when and then repeating the analyses. The Pearson correlation between fathers’ ages at birth and TRF-determined LTL was 0.11 for women and 0.14 for men (Additional file 1: Fig. S4).

Although we found a significant PAC effect, further adjusting for it in our analyses did not explain the association between longer TRF-determined LTL and increased likelihood of ART use in men in our study (Additional file 1: Fig. S15, S16 and S17). An alternative explanation might be related to common mechanistic patterns associated with male factor infertility. For instance, it has been proposed that male factor infertility, including low sperm count, may cluster within families [44, 45], potentially leading to longer TL in the sperm of fathers and their offspring’s leukocytes. This familial pattern could also contribute to the observed increased likelihood of ART use in men from such families. Further studies, with detailed analysis of male factor infertility across generations, are needed to elucidate the link between longer LTL and the higher likelihood of ART use in men.

Unmeasured confounding factors or bias may also explain the observed association between TRF-determined LTL and ART use in men [46]. Our results may have been biased by selection, as only a small number of men used ART in our study. Moreover, when we used GRS for LTL and genetically predicted LTL instead of TRF-determined LTL, the observed association between TRF-determined LTL and ART use in men was not replicated, suggesting that environmental effects on LTL that are also associated with ART use might instead be driving the association. Environmental factors potentially influencing LTL include certain lifestyle factors, such as nutrition and alcohol consumption [47]. While some studies have found significant associations between TL and nutrition and alcohol consumption [48, 49], others have failed to find such evidence [50, 51]. However, the observed associations between TRF-determined LTL and ART use in men could still reflect the influence of such unmeasured environmental factors.

Differences in TL measurement techniques may partly account for the inconsistent findings across studies. These methods encompass quantitative polymerase chain reaction (qPCR)-based approaches to various fluorescent in situ hybridization (FISH) methods and Southern blotting [52–55]. Additionally, telomere dynamics vary across different tissues, especially between germ cells and somatic cells [56]. TL can be measured in different tissues and cells, including stromal cells, leukocytes, endometrial cells, tubal epithelial cells, granulosa cells or oocytes, and sperm cells [15]. While telomeres shorten with age in most tissues, they remain relatively stable in a few tissues, including testis and ovaries, due to more robust telomerase activity [57]. Moreover, while there is considerable evidence linking oxidative stress and TL shortening in vitro, the impact of oxidative stress on TL shortening in vivo is less understood [58]. The typically small sample sizes in selected populations used in some studies may also lead to false positives. These methodological shortcomings could significantly affect the interpretation of the effect of TL on infertility-related phenotypes.

Key strengths of our study include its sample size and our ability to investigate associations with three concrete measures of LTL as opposed to using proxies: LTL assessed through direct measurements of TRFs by Southern blot, GRS constructed for LTL from GWAS summary statistics, and genetically predicted LTL. Our measurements of LTL were obtained using Southern blot, which ensures higher accuracy and directly interpretable TL in actual kilobases compared to, for example, PCR-based methods from which TL needs to be derived. We also had detailed information on TTP, a more precise and temporal measure of reproductive potential compared to for example infertility diagnoses. Moreover, we were able to investigate the risk of infertility in couples who conceived through sexual intercourse and those who conceived through ART. We also had detailed information on the reasons for using ART, which is often lacking in comparable studies.

An important limitation of our study is that MoBa is a pregnancy cohort. Since all participants were recruited based on having achieved a pregnancy, we were unable to investigate associations in women and men with the most severe infertility problems, such as those who never conceived or experienced early pregnancy loss. Importantly, LTL may be a more appropriate biomarker for reproductive traits in childless women and men with infertility, a hypothesis worth exploring in future studies. Given the lack of data on childless women and men, we were also unable to investigate the probability of using ART independent of treatment outcome. Furthermore, recall bias may have influenced our findings, particularly due to potential inaccuracies in participants’ recollections of their TTP. Although previous research has shown that TTPs of less than 12 months are generally well-recalled when reported retrospectively during pregnancy [59], the longer TTPs in our fecundability analyses may have been more susceptible to recall bias. Lastly, MoBa is generally a selected and homogeneous group of individuals, representing women and men within a higher socioeconomic bracket compared to the population at large.

## Conclusions

In conclusion, we found no significant evidence that LTL measures influence fecundability, infertility, or use of ART in either women or men. An exception was a modest association observed between longer TRF-determined LTL and a higher likelihood of ART use in men. Because this association was not observed when examining the same relationships using the other two measures of LTL (GRS for LTL and genetically-predicted LTL), it may be that unmeasured environmental factors could be related to both LTL and ART use in men. Overall, there was no evidence supporting the use of LTL as a biomarker for assessing fecundability, infertility, or ART use. However, this null finding warrants validation in other large cohorts with comparable data, and which also include couples who did not conceive as a control group.

## Supplementary information

Additional file 1. Figs. S1-S17. Fig S1. Age distributions among participants. Fig S2. Correlation between telomere length and age. Fig S3. Correlation between telomere length in women and men. Fig S4. Paternal age effects. Fig S5. Correlation between telomere length and genetic risk scores for telomere length. Fig S6. Correlation between telomere length and genetically predicted telomere length. Fig. S7. Within-cycle probabilities of conception for participants with telomere data. Fig. S8. Within-cycle probabilities of conception for participants with genotype data. Fig. S9. Telomere length and fecundability in age-restricted sample. Fig. S10. Telomere length and infertility in age-restricted sample. Fig. S11. Telomere length and use of ART in age-restricted sample. Fig. S12. Telomere length and fecundability adjusted for partners’ telomere length. Fig. S13. Telomere length and infertility adjusted for partners’ telomere length. Fig. S14. Telomere length and use of ART adjusted for partners’ telomere length. Fig. S15. Telomere length and fecundability adjusted for PAC effects. Fig. S16. Telomere length and infertility adjusted for PAC effects. Fig. S17. Telomere length and use of ART adjusted for PAC effects. Additional file 1: Tables S1-S3. Table S1. Characteristics of the study population. Table S2. Robustness of genetic risk scores. Table S3. Statistical tests for non-linear associations.

## Data Availability

No datasets were generated or analysed during the current study.

## Abbreviations

2SLS: Two-stage least squares
AIC: Akaike information criterion
ART: Assisted reproductive technologies
BMI: Body mass index
CI: Confidence interval
EDF: Effective degrees of freedom
FISH: Fluorescent in situ hybridization
FR: Fecundability ratio
GAM: Generalized additive model
GRS: Genetic risk score
GWAS: Genome-wide association study
HRC: The European Genome-Phenome Archive Haplotype Reference Consortium
LTL: Leukocyte telomere length
MAF: Minor allele frequency
MBRN: The Medical Birth Registry of Norway
MoBa: The Norwegian Mother, Father, and Child Cohort Study
OR: Odds ratio
PAC: Paternal age at conception
PCOS: Polycystic ovary syndrome
QC: Quality control
qPCR: Quantitative polymerase chain reaction
SD: Standard deviation
SNP: Single-nucleotide polymorphism
STROBE: Strengthening the Reporting of Observational Studies in Epidemiology
TL: Telomere length
TRF: Terminal restriction fragment
TTP: Time to pregnancy
