# Supplementary material for: Telomere length in relation to fecundability and use of assisted reproductive technologies: the Norwegian Mother, Father, and Child Cohort Study
Source: BMC Med. 2024 Dec 18;22:580. doi: 10.1186/s12916-024-03795-0 (PMC11658396; doi:10.1186/s12916-024-03795-0)
Supplement: Supplementary file 1 — Additional file 1. Figs. S1-S17. Fig S1. Age distributions among participants. Fig S2. Correlation between telomere length and age. Fig S3. Correlation between telomere length in women and men. Fig S4. Paternal age effects. Fig S5. Correlation between telomere length and genetic risk scores for telomere length. Fig S6. Correlation between telomere length and genetically predicted telomere length. Fig. S7. Within-cycle probabilities of conception for participants with telomere data. Fig. S8. Within-cycle probabilities of conception for participants with genotype data. Fig. S9. Telomere length and fecundability in age-restricted sample. Fig. S10. Telomere length and infertility in age-restricted sample. Fig. S11. Telomere length and use of ART in age-restricted sample. Fig. S12. Telomere length and fecundability adjusted for partners’ telomere length. Fig. S13. Telomere length and infertility adjusted for partners’ telomere length. Fig. S14. Telomere length and use of ART adjusted for partners’ telomere length. Fig. S15. Telomere length and fecundability adjusted for PAC effects. Fig. S16. Telomere length and infertility adjusted for PAC effects. Fig. S17. Telomere length and use of ART adjusted for PAC effects. Additional file 1: Tables S1-S3. Table S1. Characteristics of the study population. Table S2. Robustness of genetic risk scores. Table S3. Statistical tests for non-linear associations. [file 12916_2024_3795_MOESM1_ESM.docx]

**Additional File 1: Supplementary Materials**

**Contents:**

Supplementary Figures

Fig. S1: Age distributions among participants.

Fig. S2: Correlation between telomere length and age.

Fig. S3: Correlation between telomere length in women and men.

Fig. S4: Paternal age effects.

Fig. S5: Correlation between telomere length and genetic risk scores for telomere length.

Fig. S6: Correlation between telomere length and genetically predicted telomere length.

Fig. S7: Within-cycle probabilities of conception for participants with telomere data.

Fig. S8: Within-cycle probabilities of conception for participants with genotype data.

Fig. S9: Telomere length and fecundability in age-restricted sample.

Fig. S10: Telomere length and infertility in age-restricted sample.

Fig. S11: Telomere length and use of ART in age-restricted sample.

Fig. S12: Telomere length and fecundability adjusted for partners’ telomere length.

Fig. S13: Telomere length and infertility adjusted for partners’ telomere length.

Fig. S14: Telomere length and use of ART adjusted for partners’ telomere length.

Fig. S15: Telomere length and fecundability adjusted for paternal age at conception effects.

Fig. S16: Telomere length and infertility adjusted for paternal age at conception effects.

Fig. S17: Telomere length and use of ART adjusted for paternal age at conception effects.

Supplementary Tables

Tab. S1: Characteristics of the study population.

Tab. S2: Robustness of genetic risk scores.

Table S3: Statistical tests for non-linear associations.


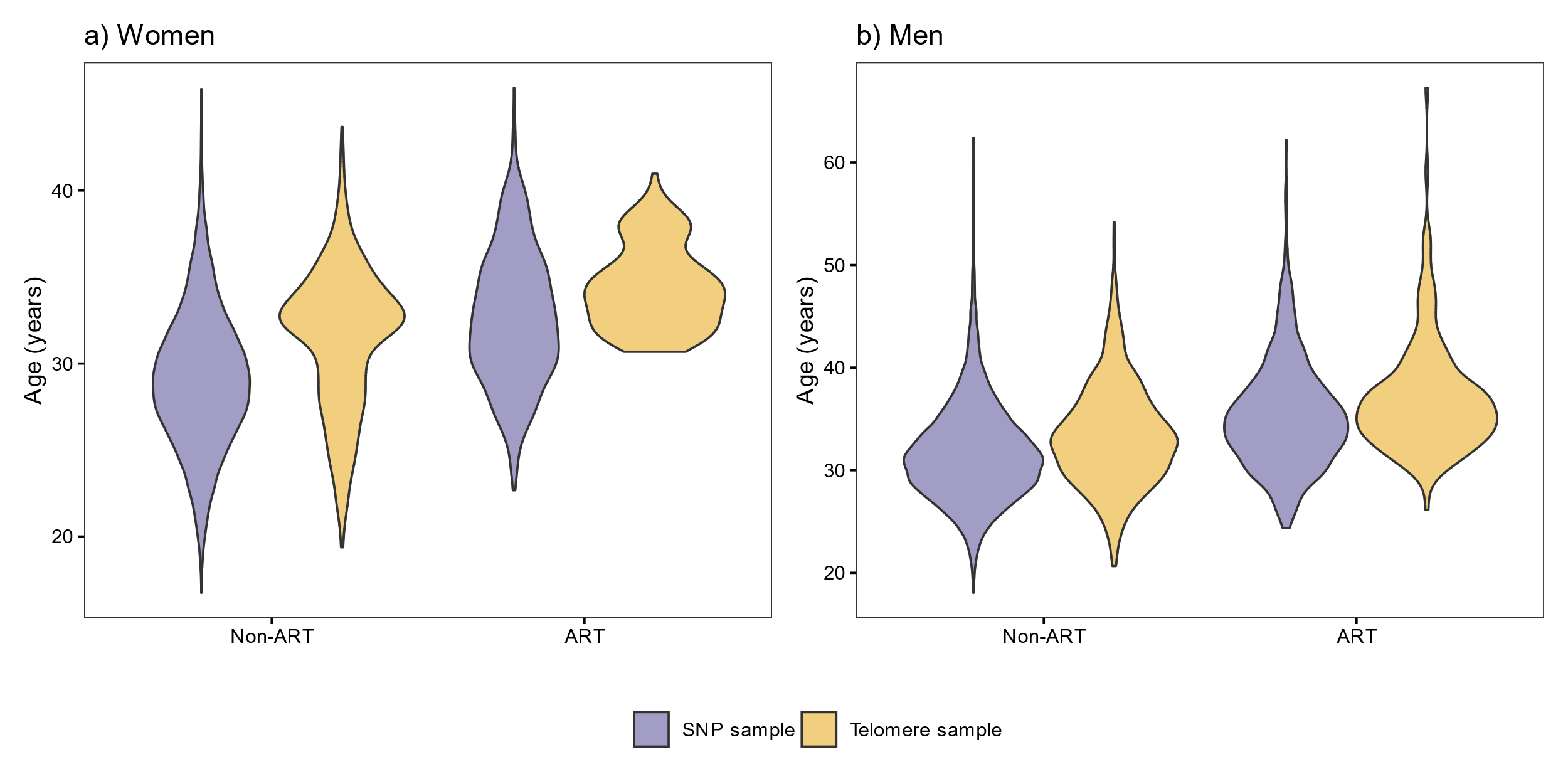


**Figure S1: Age distributions among participants.** Density estimates of age within assisted reproductive technologies (ART) users and non-ART users for the SNP sample (yellow) and telomere sample (purple) in a) women and b) men.


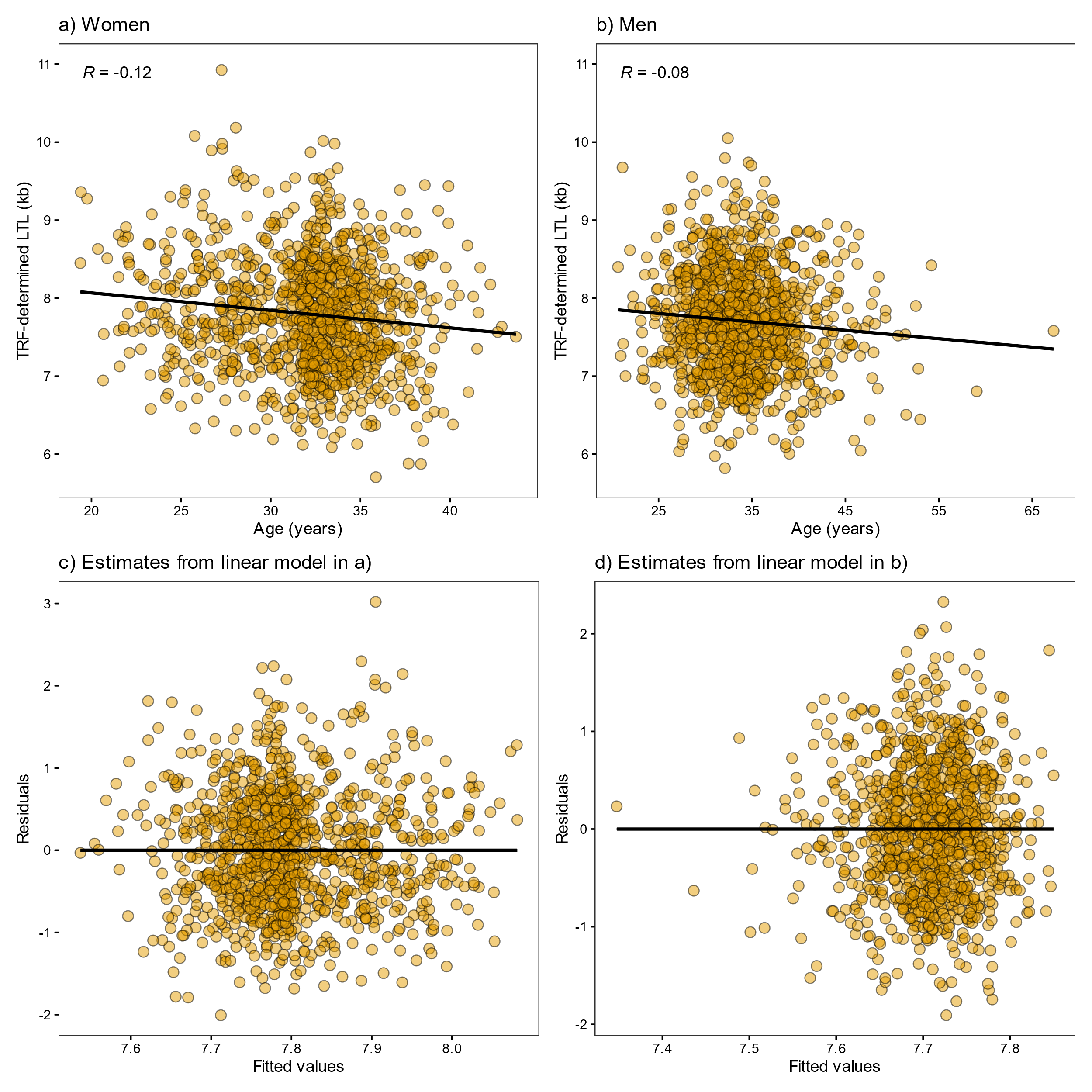


**Figure S2: Correlation between telomere length and age.** TRF-determined leukocyte telomere length (LTL) distribution across chronological age in a) women and b) men. Fitted values and residuals from the linear model in a) in c) and for b) in d).


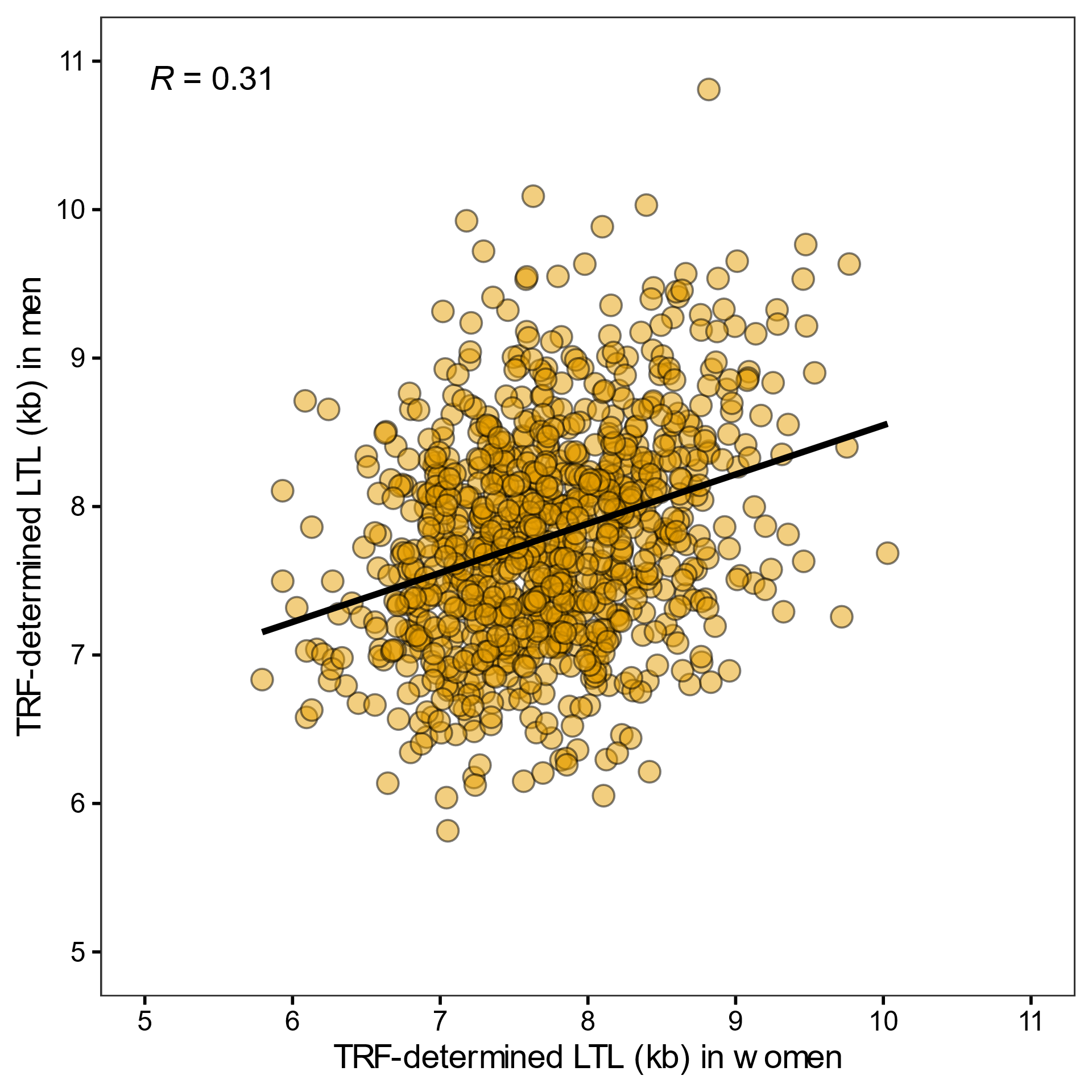


**Figure S3: Correlation between telomere length in women and men.** Correlation between age-adjusted TRF-determined leukocyte telomere length (LTL) in women and age-adjusted TRF-determined LTL in men within couples. The Pearson correlation between female and male LTL is presented in the top left corner.


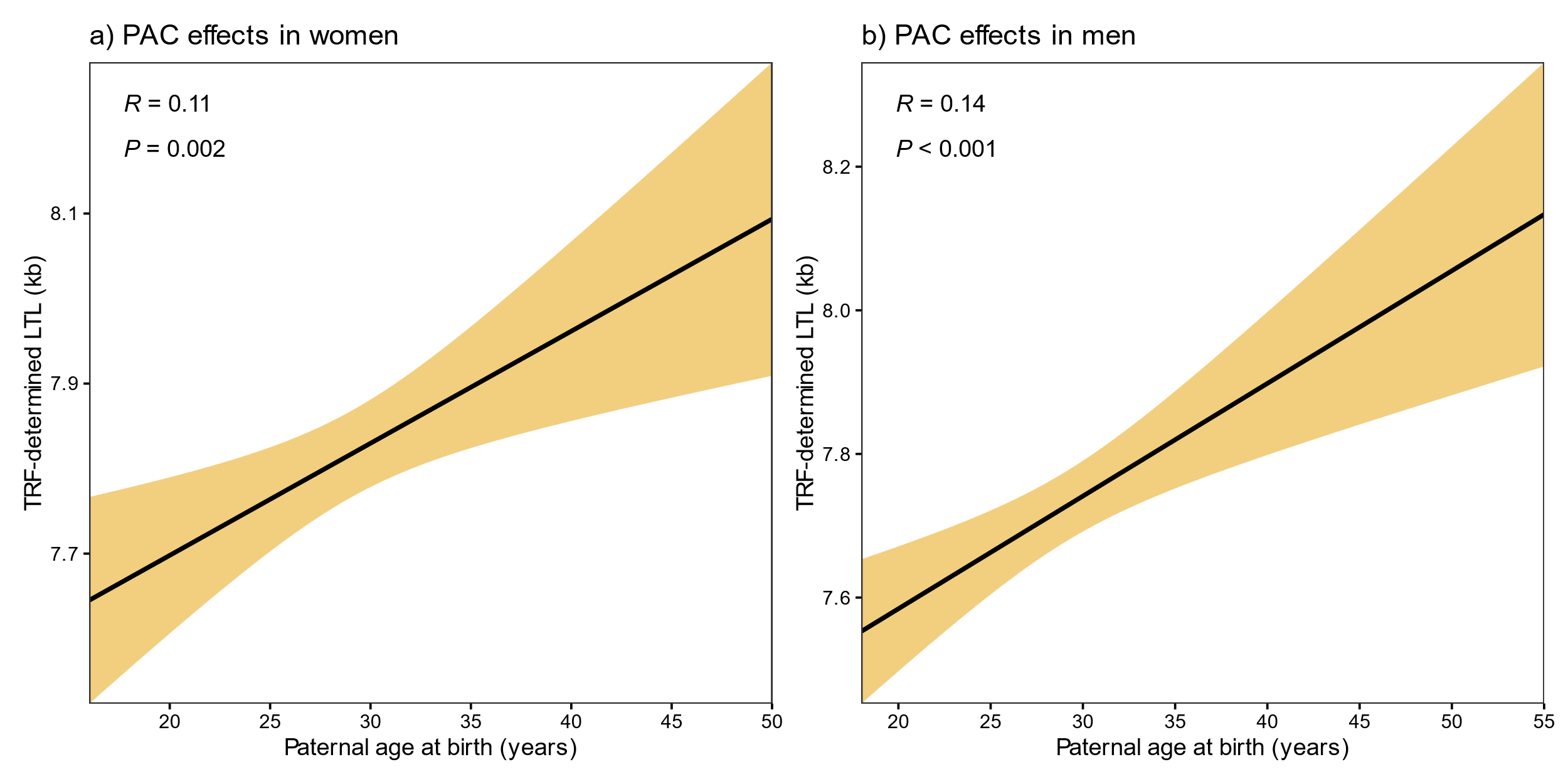


**Figure S4: Paternal age effects.** Association between paternal age at conception (PAC) effects and age-adjusted TRF-determined leukocyte telomere length (LTL) in a) women and b) and men.


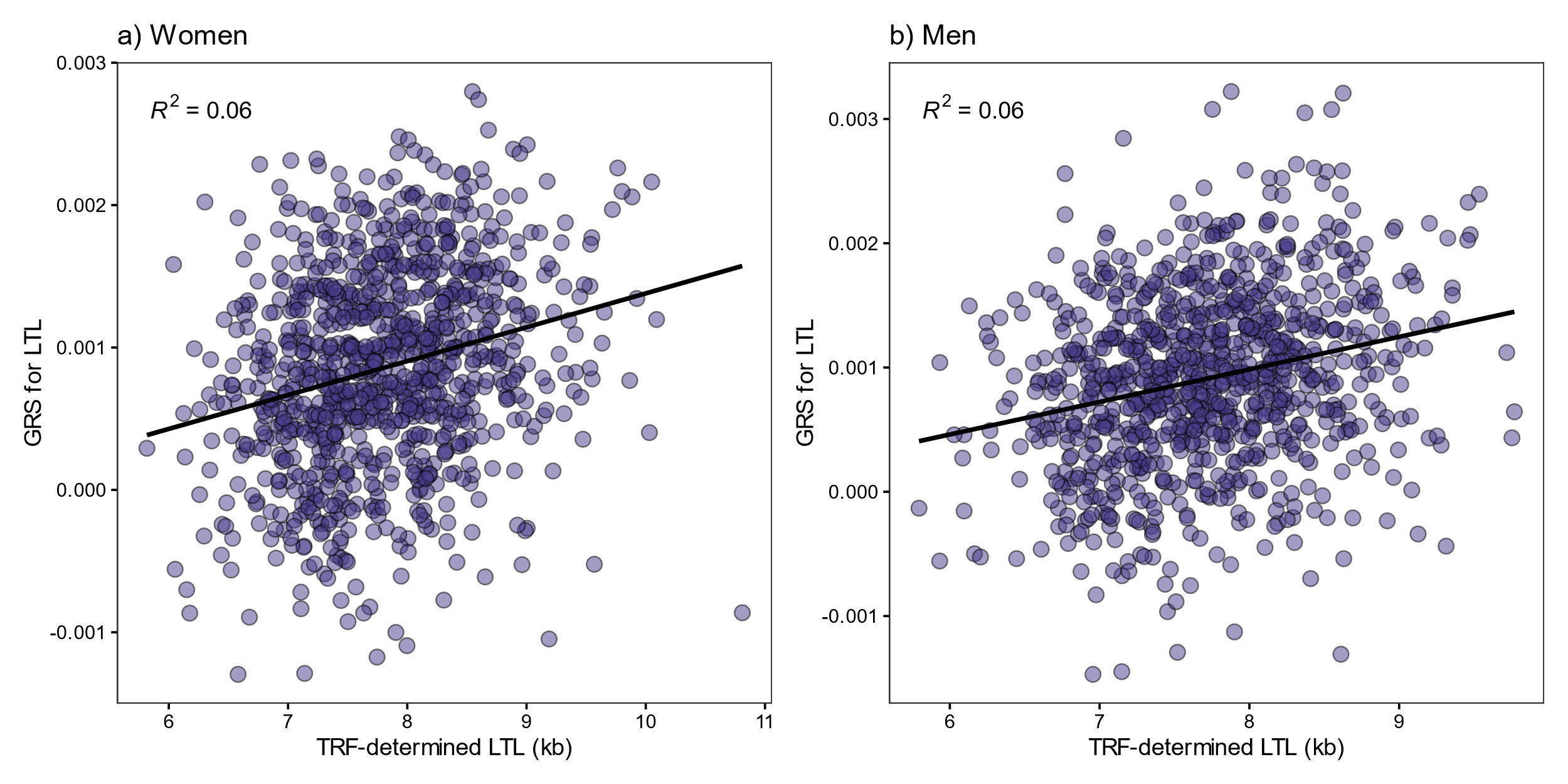


**Figure S5: Correlation between telomere length and genetic risk scores for telomere length.** Correlation between age-adjusted TRF-determined leukocyte telomere length (LTL) and genetic risk scores (GRS) for LTL in a) women and b) men.


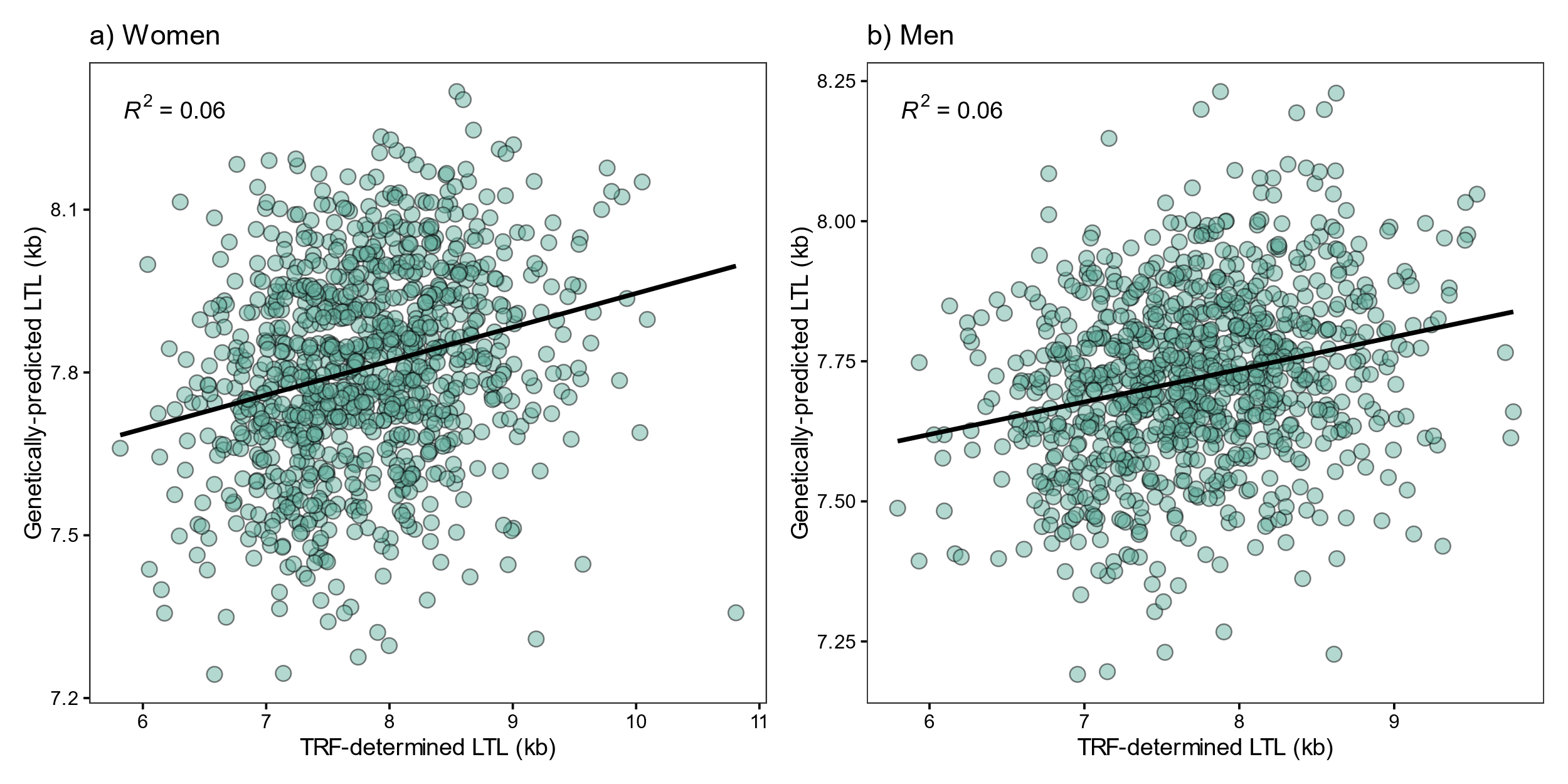


**Figure S6: Correlation between telomere length and genetically predicted telomere length.** Correlation between age-adjusted TRF-determined leukocyte telomere length (LTL) and genetically-predicted LTL in a) women and b) men.


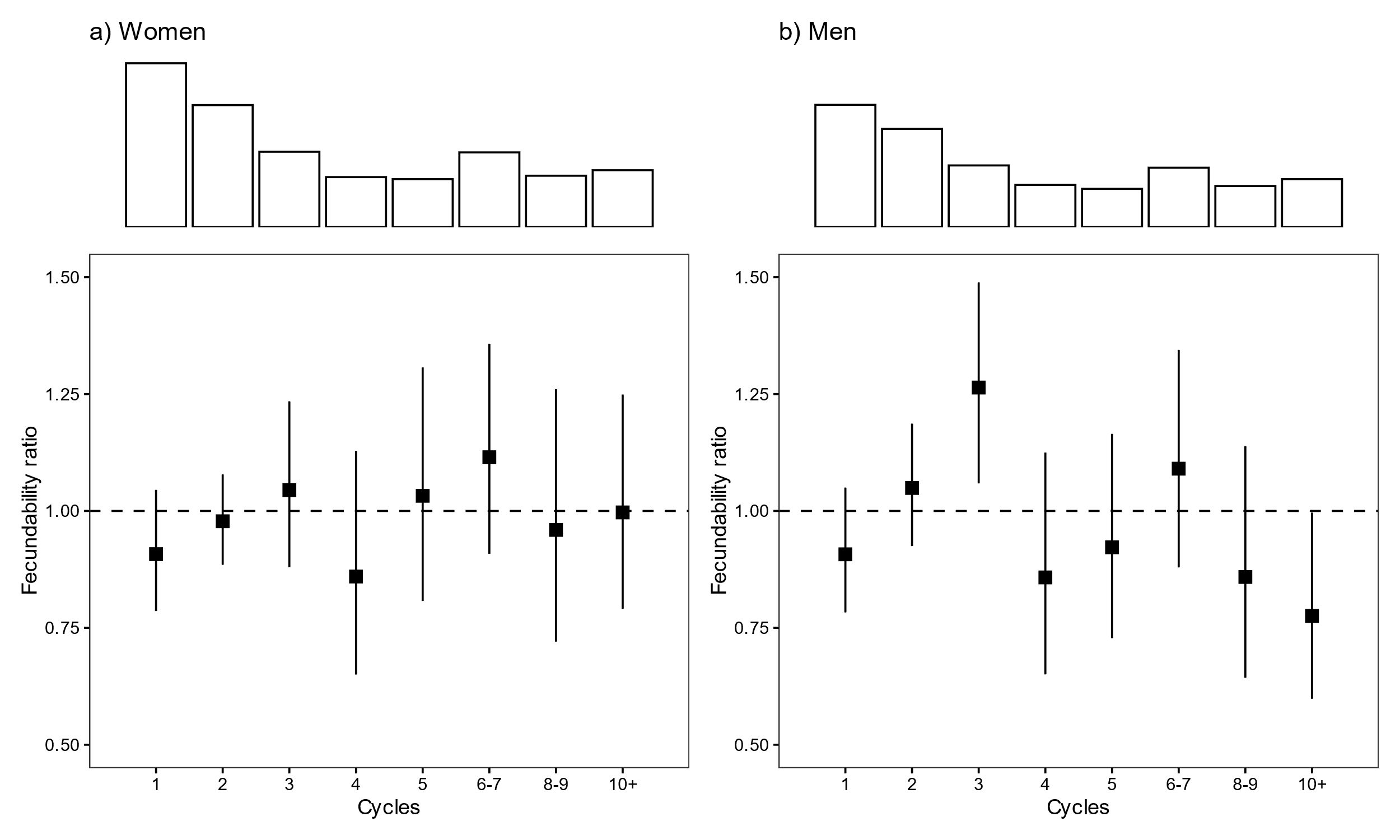


**Figure S7: Within-cycle probabilities of conception for participants with telomere data.** The probability to conceive within each cycle (FR; fecundability ratio) for the sample of TRF-determined leukocyte telomere length (LTL). Due to low sample sizes within the cycles after the 5^th^ cycle, we collapsed these cycles into groups of 6-7 cycles, 8-9 cycles and 10 or more cycles. The distribution of women and men conceiving within each cycle is presented on top.


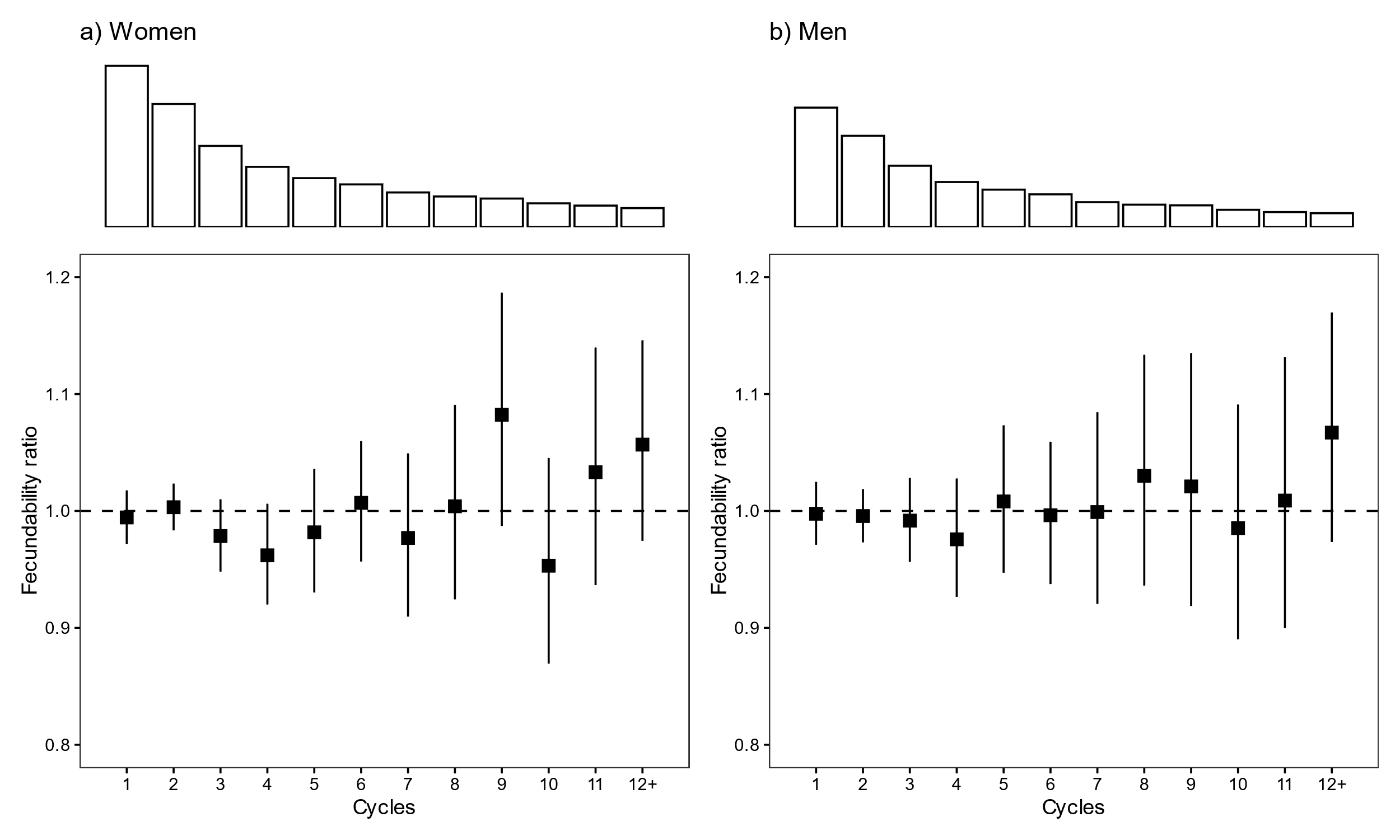


**Figure S8:** **Within-cycle probabilities of conception for participants with genotype data.** The probability to conceive within each cycle (FR; fecundability ratio) for the sample of genetic risk scores (GRS) for leukocyte telomere length (LTL). The distribution of women and men conceiving within each cycle is presented on top.


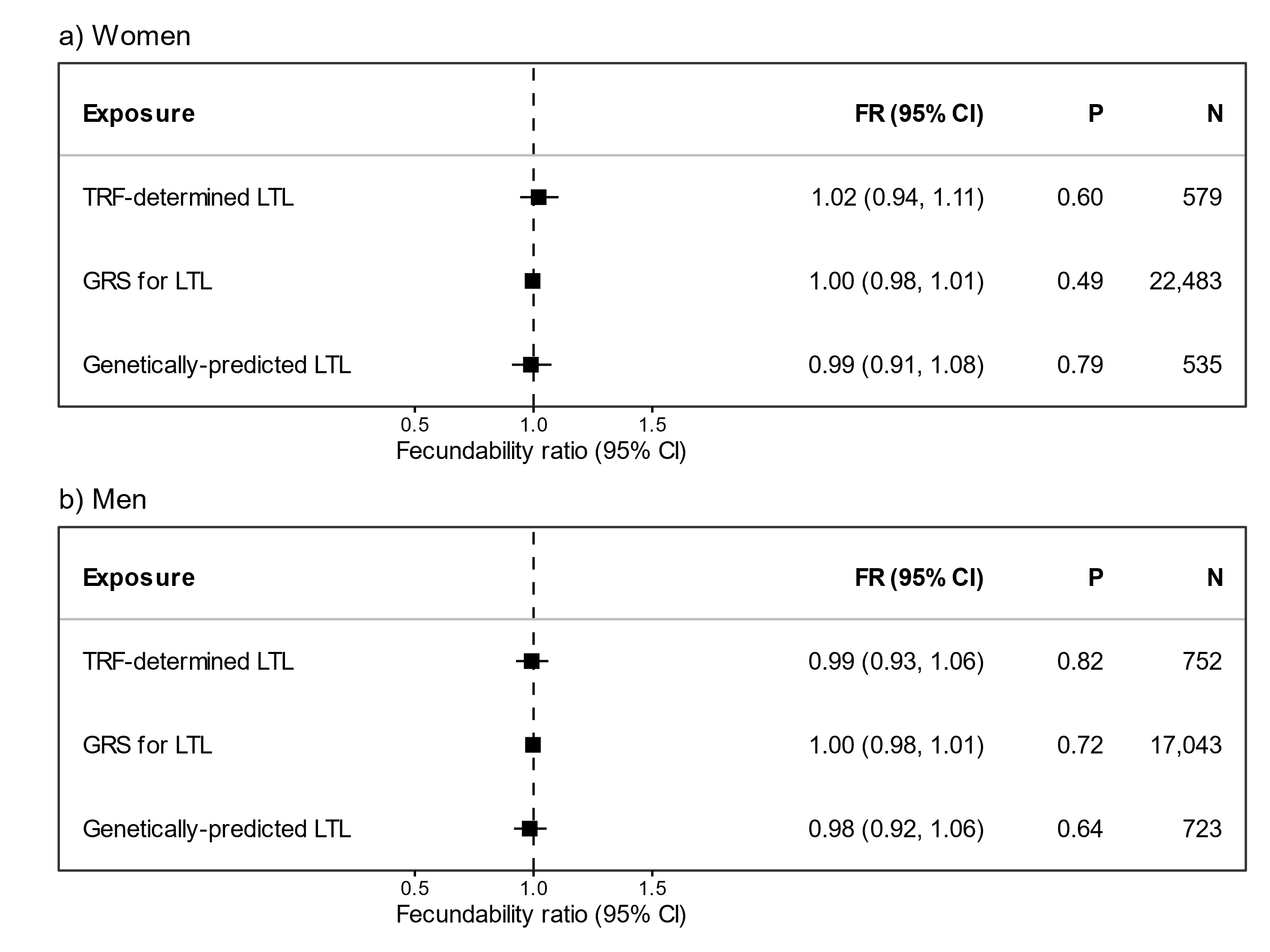


**Figure S9: Telomere length and fecundability in age-restricted sample.** The association between a standard deviation (SD) increase of leukocyte telomere length (LTL) exposures and fecundability in a) women and b) men restricting to women aged 30 years or older and their partners.

**
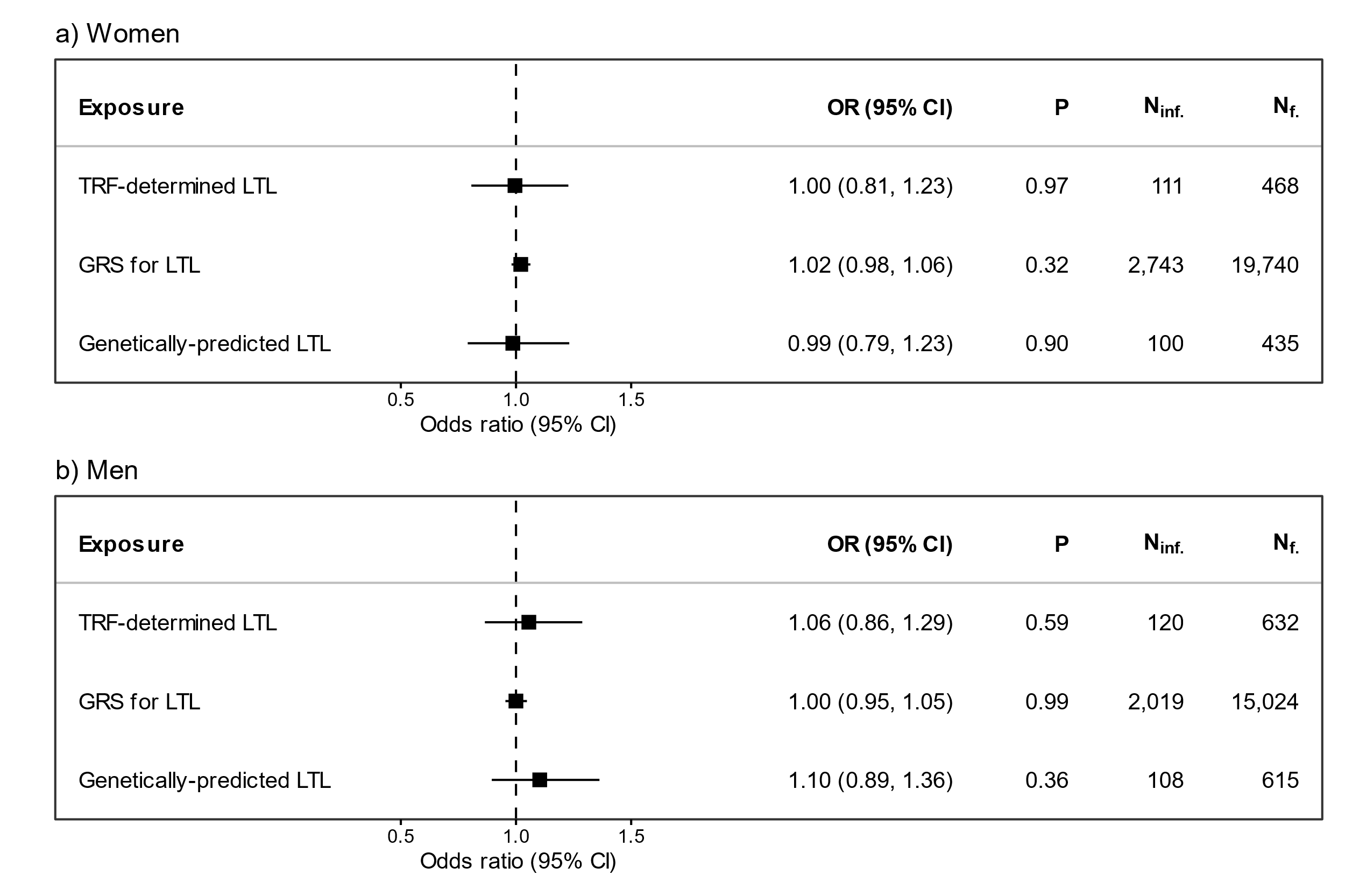
**

**Figure S10:** **Telomere length and infertility in age-restricted sample.** The association between a standard deviation (SD) increase of leukocyte telomere length (LTL) exposures and infertility in a) women and b) men restricting to women aged 30 years or older and their partners.


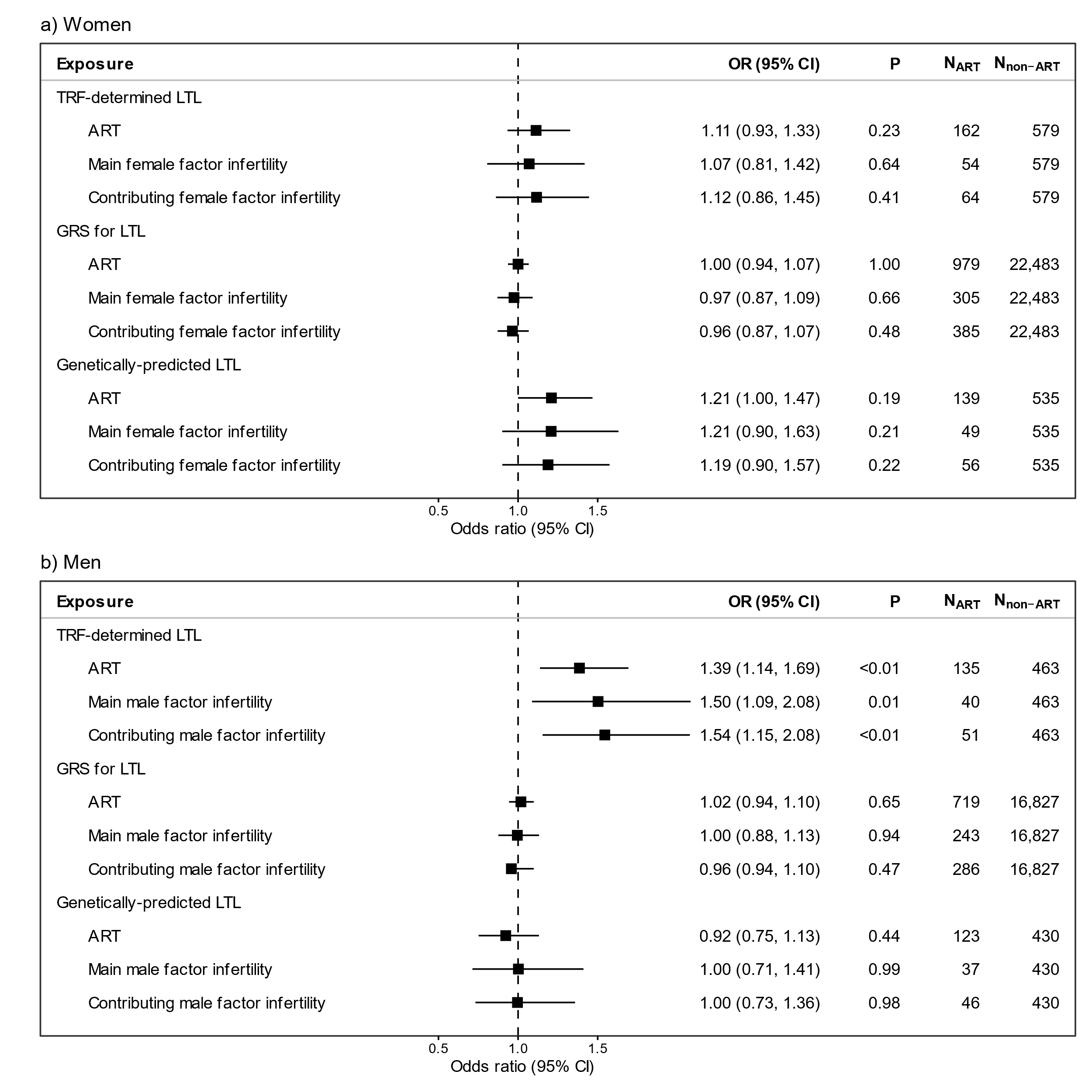


**Figure S11: Telomere length and use of ART in age-restricted sample.** The association between a standard deviation (SD) increase of leukocyte telomere length (LTL) exposures and having conceived through assisted reproductive technologies (ART) in a) women and b) men restricting to women aged 30 years or older and their partners.


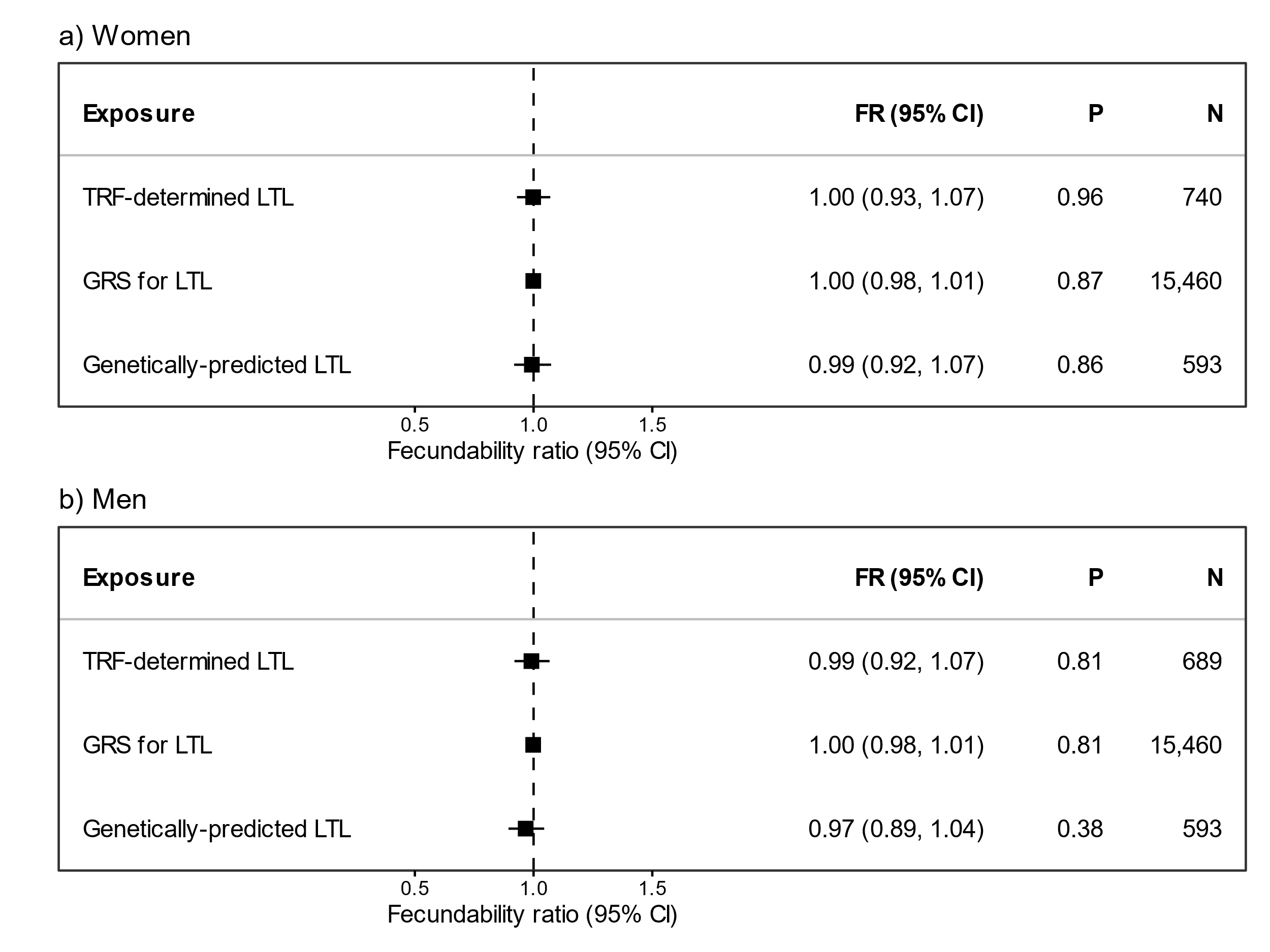


**Figure S12: Telomere length and fecundability adjusted for partners’ telomere length.** The association between a standard deviation (SD) increase of leukocyte telomere length (LTL) exposures and fecundability in a) women and b) men mutually adjusting for partners’ LTL.


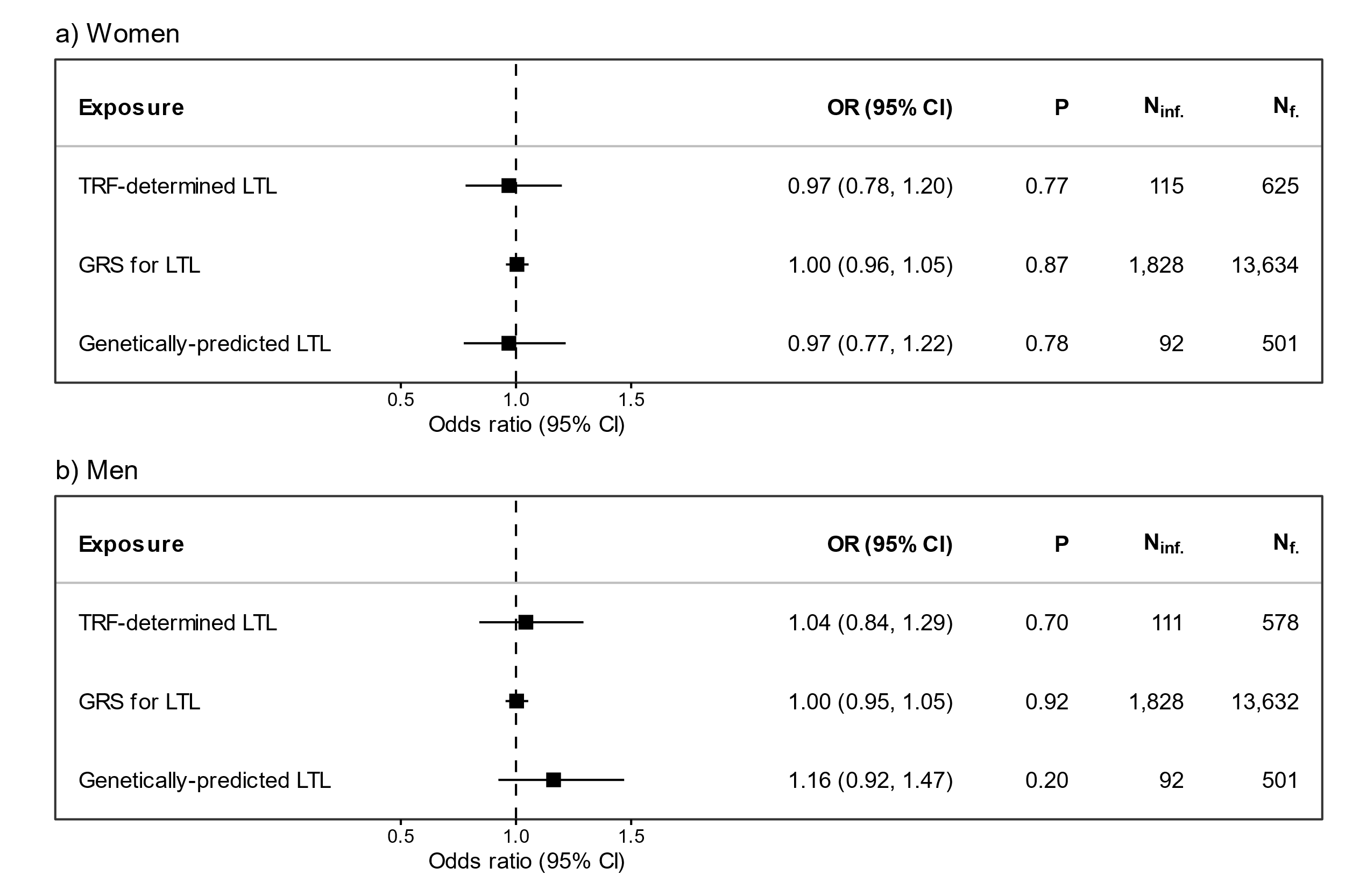


**Figure S13: Telomere length and infertility adjusted for partners’ telomere length.** The association between a standard deviation (SD) increase of leukocyte telomere length (LTL) exposures and infertility in a) women and b) men mutually adjusting for partners’ LTL.


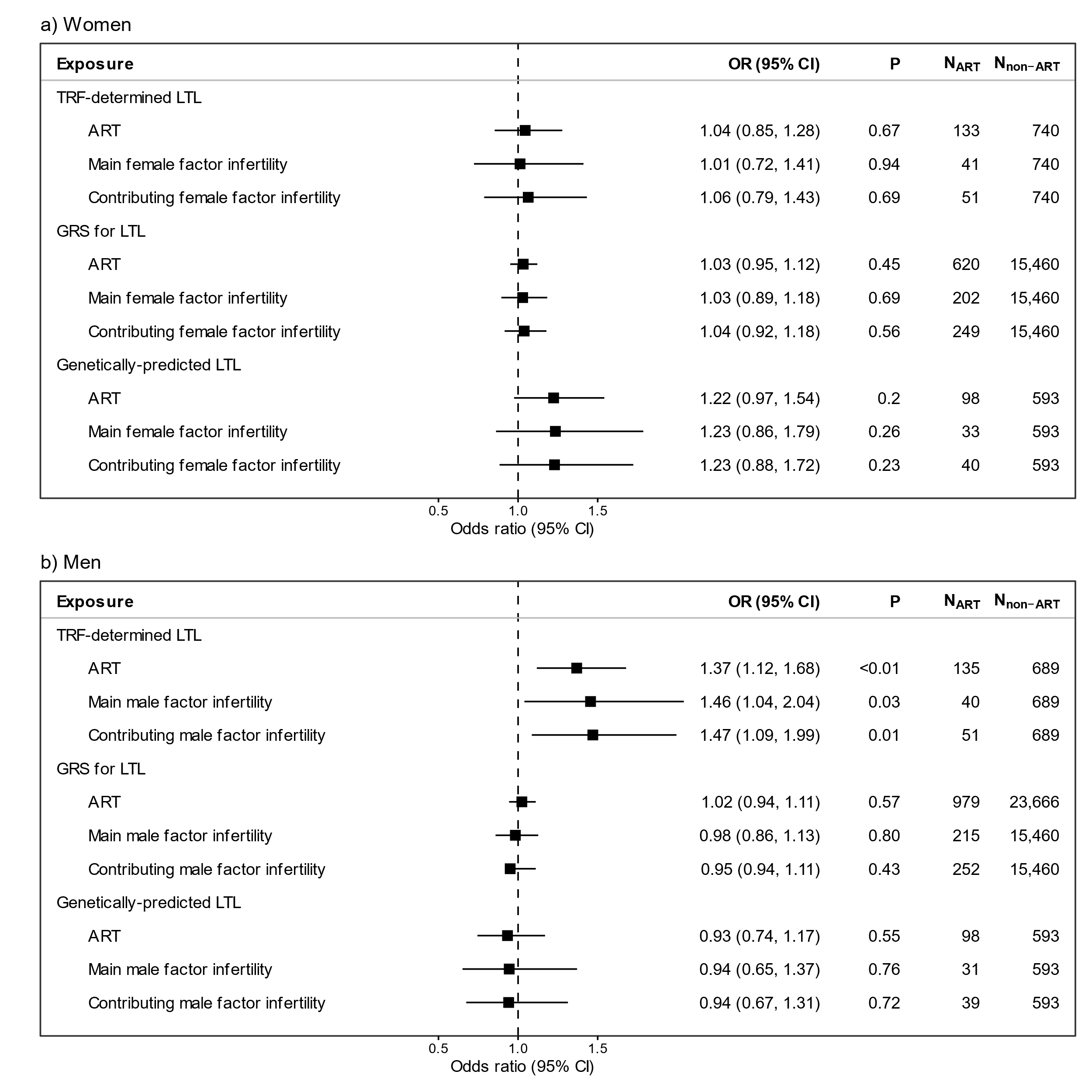


**Figure S14: Telomere length and use of ART adjusted for partners’ telomere length.** The association between a standard deviation (SD) increase of leukocyte telomere length (LTL) exposures and having conceived through assisted reproductive technologies (ART) in a) women and b) men mutually adjusting for partners’ LTL.


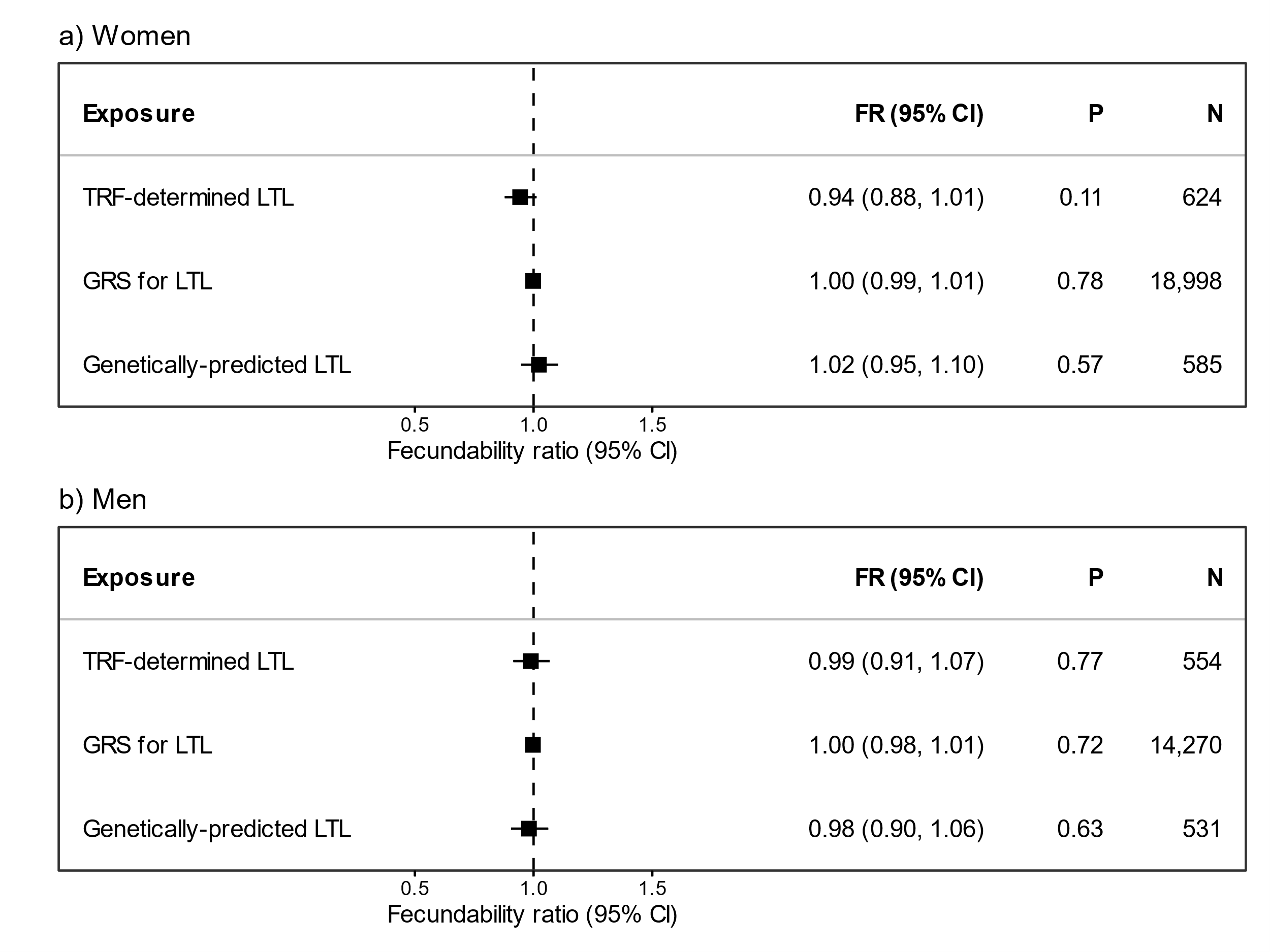


**Figure S15: Telomere length and fecundability adjusted for paternal age at conception effects.** The association between a standard deviation (SD) increase of leukocyte telomere length (LTL) exposures and fecundability in a) women and b) men. TRF-determined LTL and genetically-predicted LTL are adjusted for paternal age at conception (PAC) effects.


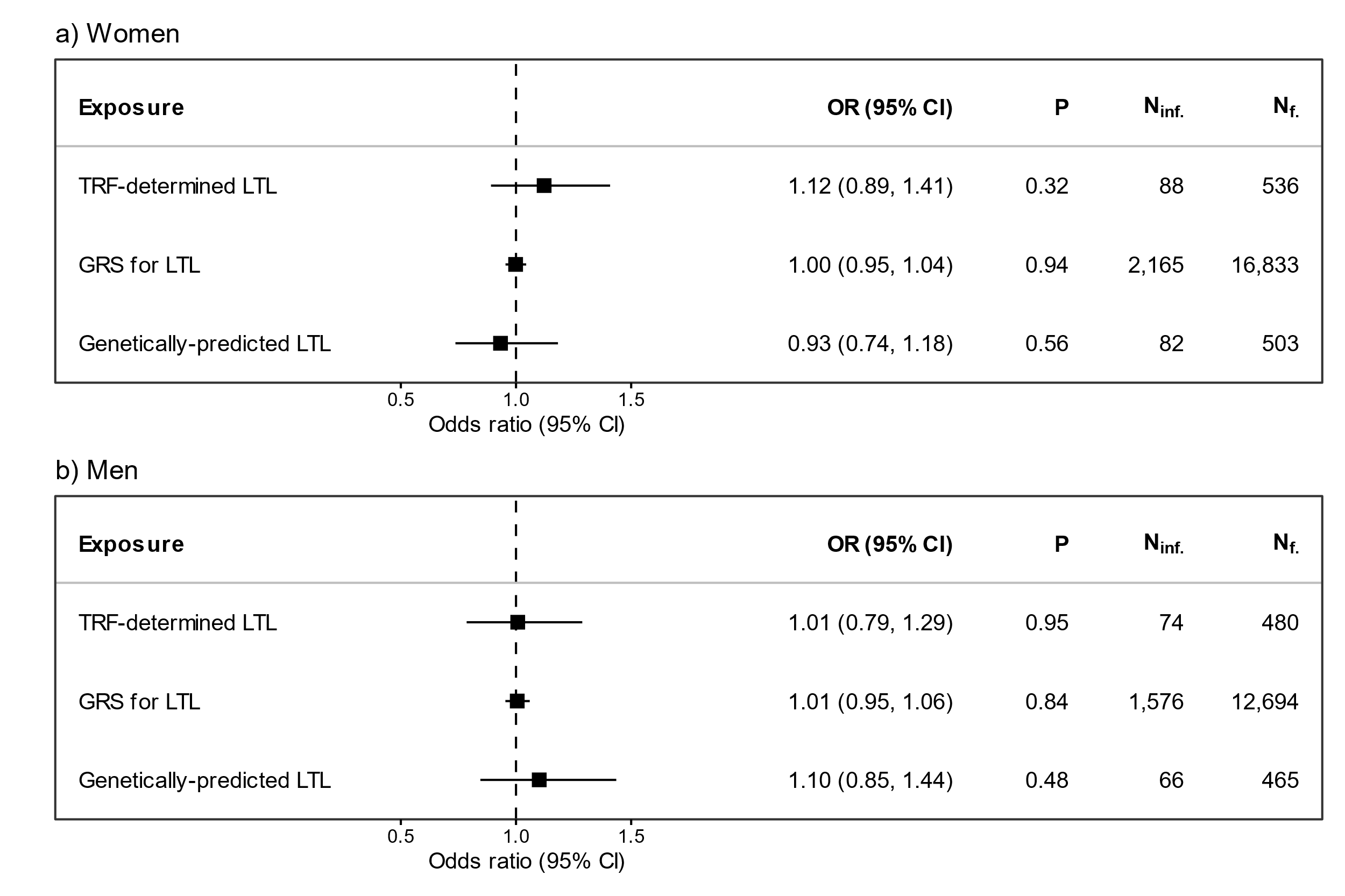


**Figure S16: Telomere length and infertility adjusted for paternal age at conception effects.** The association between a standard deviation (SD) increase of leukocyte telomere length (LTL) exposures and infertility in a) women and b) men. TRF-determined LTL and genetically-predicted LTL are adjusted for paternal age at conception (PAC) effects.


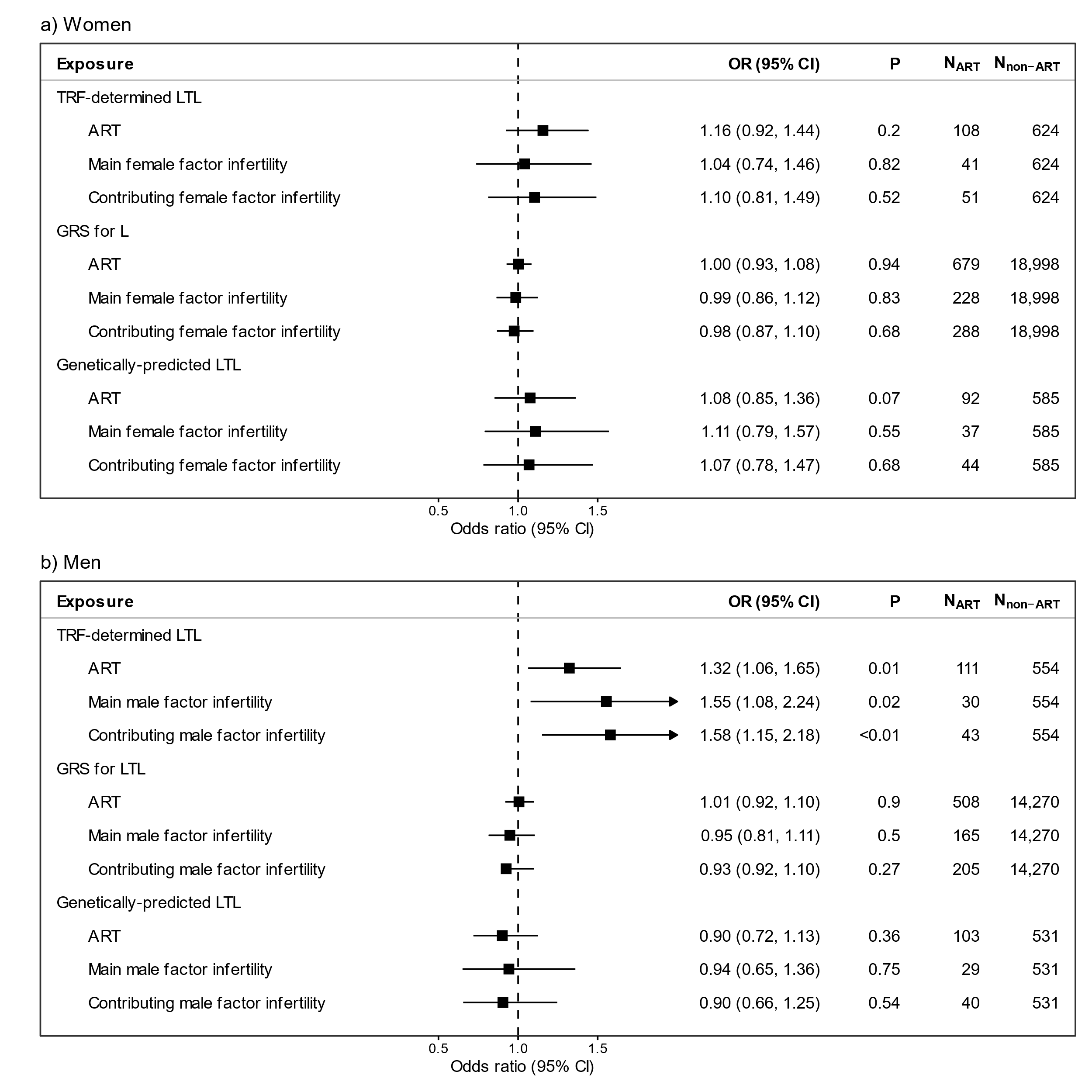


**Figure S17: Telomere length and use of ART adjusted for paternal age at conception effects.** The association between a standard deviation (SD) increase of leukocyte telomere length (LTL) exposures and having conceived through assisted reproductive technologies (ART) in a) women and b) men. TRF-determined LTL and genetically-predicted LTL are adjusted for paternal age at conception (PAC) effects.

**Table S1: Characteristics of the study population.** Characteristics of the sample with genetic data within our study population.

|  | **Women** | | | **Men** | | |
| --- | --- | --- | --- | --- | --- | --- |
|  | **Fertile** | **Infertile** | **ART** | **Fertile** | **Infertile** | **ART** |
| N (%) | 20,629 | 3,037 | 979 | 15,400 | 2,220 | 719 |
| Age, median (IQR) | 28.9 (26.5, 31.5) | 30.2 (27.3, 33.2) | 32.8 (30.3, 35.5) | 31.1 (28.5, 34.1) | 32.5 (29.5, 35.8) | 35.1 (32.3, 38.2) |
| TTP, median (IQR) | 2 (1, 4) | 17 (13, 24) |  | 2 (1, 4) | 17 (12, 24) |  |
| BMI, median (IQR) | 22.7 (20.9, 25.1) | 23.3 (21.1, 26.5) | 23.0 (21.2, 25.7) | 25.2 (23.5, 27.4) | 25.6 (23.8, 28.1) | 25.6 (24.0, 27.8) |
| BMI, N (%) | 20,287 (98.3) | 3,002 (98.9) | 913 (93.3) | 15,165 (98.5) | 2,172 (97.8) | 676 (94.0) |
| Missing, N (%) | 342 (1.7) | 35 (1.2) | 66 (6.7) | 237 (1.5) | 48 (2.2) | 43 (5.6) |
| Higher education, N (%) | 14,747 (71.5) | 1,955 (64.4) | 678 (69.3) | 8,755 (56.9) | 1,105 (49.8) | 415 (57.7) |
| Lower education, N (%) | 5,467 (26.5) | 1,019 (33.6) | 227 (23.2) | 6,411 (41.6) | 1,075 (48.14) | 258 (35.9) |
| Missing, N (%) | 415 (2.0) | 63 (2.1) | 74 (7.6) | 234 (1.5) | 40 (1.8) | 46 (6.4) |
| Non-smoker, N (%) | 10,505 (50.9) | 1,419 (46.7) | 482 (49.2) | 6,246 (40.6) | 868 (39.1) | 280 (38.9) |
| Former smoker, N (%) | 3,882 (18.8) | 558 (18.4) | 236 (24.1) | 4,295 (27.9) | 621 (28.0) | 203 (28.2) |
| Smoker, N (%) | 5,867 (28.5) | 1,012 (33.3) | 214 (21.9) | 4,309 (27.9) | 660 (29.7) | 178 (24.8) |
| Missing, N (%) | 375 (1.8) | 48 (1.6) | 47 (4.8) | 550 (3.6) | 71 (3.2) | 58 (8.1) |

Abbreviations: ART, assisted reproductive technologies; TTP, time to pregnancy; BMI, body mass index.

**Table S2: Robustness of genetic risk scores.** Robustness of genetic risk scores (GRS) for age-adjusted leukocyte telomere length (LTL).

|  | **N** | **F-statistic** | **P-value** | **Adjusted R^2^** |
| --- | --- | --- | --- | --- |
| Women | 144 | 61 | <0.05 | 0.06 |
| Men | 144 | 60 | <0.05 | 0.06 |

**Table S3: Statistical tests for non-linear associations.**

|  | **Women** | | | | | **Men** | | | | |
| --- | --- | --- | --- | --- | --- | --- | --- | --- | --- | --- |
|  | **Linear model** | | **Non-linear model** | | | **Linear model** | | **Non-linear model** | | |
|  | **AIC** | **P-value** | **AIC** | **P-value** | **EDF^a^** | **AIC** | **P-value** | **AIC** | **P-value** | **EDF^a^** |
| TRF-determined LTL | 3,960.2 | 0.52 | 3,960.2 | 0.59 | 1.08 | 3,614.2 | 0.82 | 3,615.3 | 0.23 | 8.02 |
| GRS for LTL | 99,689.3 | 0.43 | 99,685.7 | 0.49 | 1.02 | 74,240.2 | 0.71 | 74,240.0 | 0.73 | 1.02 |
| Genetically-predicted LTL | 3,645.3 | 0.77 | 3,643.7 | 0.03 | 4.98 | 3,445.3 | 0.36 | 2,957.6 | 0.42 | 1.66 |

Abbreviations: TL, telomere length; AIC, Akaike information criterion; EDF, effective degrees of freedom.

^a^ An EDF of 1.0 implies a linear association.
